# Environmental factors shaping stable isotope signatures of modern red deer (*Cervus elaphus*) inhabiting various habitats

**DOI:** 10.1371/journal.pone.0255398

**Published:** 2021-08-13

**Authors:** Maciej Sykut, Sławomira Pawełczyk, Tomasz Borowik, Boštjan Pokorny, Katarina Flajšman, Tjibbe Hunink, Magdalena Niedziałkowska

**Affiliations:** 1 Mammal Research Institute, Polish Academy of Sciences, Białowieża, Poland; 2 Division of Geochronology and Environmental Isotopes, GADAM Centre of Excellence, Institute of Physics, Center for Science and Education, Silesian University of Technology, Gliwice, Poland; 3 Environmental Protection College, Velenje, Slovenia; 4 Slovenian Forestry Institute, Ljubljana, Slovenia; 5 Staatsbosbeheer / Flevoland, Lelystad, The Netherlands; Senckenberg Gesellschaft fur Naturforschung, GERMANY

## Abstract

Stable isotope analyses of bone collagen are often used in palaeoecological studies to reveal environmental conditions in the habitats of different herbivore species. However, such studies require valuable reference data, obtained from analyses of modern individuals, in habitats of well-known conditions. In this article, we present the stable carbon and nitrogen isotope composition of bone collagen from modern red deer (N = 242 individuals) dwelling in various habitats (N = 15 study sites) in Europe. We investigated which of the selected climatic and environmental factors affected the *δ*^13^C and *δ*^15^N values in bone collagen of the studied specimens. Among all analyzed factors, the percent of forest cover influenced the carbon isotopic composition most significantly, and decreasing forest cover caused an increase in *δ*^13^C values. The *δ*^15^N was positively related to the proportion of open area and (only in the coastal areas) negatively related to the distance to the seashore. Using rigorous statistical methods and a large number of samples, we confirmed that *δ*^13^C and *δ*^15^N values can be used as a proxy of past habitats of red deer.

## 1. Introduction

The analyses of stable isotopes of carbon and nitrogen in bone collagen of ungulate remains is frequently used in palaeoenvironmental and palaeoclimatic studies [1 and references therein]. It is well documented that the isotope signatures of plants are transferred up the food chain to herbivores and are recorded in their tissues [2]. Since the turnover rate of bone collagen (i.e., the rate at which the elements the tissue is composed of are replaced during the lifetime) is relatively slow, the isotopic composition of bone collagen reflects time-averaged information from the last few years of the animal’s life [3]. Due to long-term bone collagen preservation, even up to ~3.4 Ma in low temperatures of the permafrost [4], the analyses of stable isotopes of carbon (*δ*^13^C) and nitrogen (*δ*^15^N) in bone collagen of ungulates have been applied in palaeoecological studies to reveal their diet, foraging habits, and habitat use [5–7]. However, climatic and environmental factors such as precipitation, temperature, salinity, and altitude may influence stable carbon and nitrogen composition of plants [8–13]; therefore, isotopic composition analyses of herbivore tissues in such palaeoecological reconstructions are challenging. On the other hand, the *δ*^13^C values of vegetation clearly reflect the contrast between open and forested environments associated with the “canopy cover effect”—change along a vertical gradient of forest trees and plants, with higher *δ*^13^C values at the top of the canopy and lower ones on the forest floor. The “canopy cover effect” is thereby reflected in a ^13^C depletion in plants growing under the canopy of dense forest stands compared with those grown in open habitats [e.g., 14]. Since herbivore tissues record the *δ*^13^C values of their plant food, it is possible to identify the type of environment in which the studied animals fed: closed (forested) or open habitats [e.g., 15]. Plant *δ*^15^N values can vary according to the same abiotic factors as plant *δ*^13^C values [16–20] and also differ among groups of plants. For example, higher *δ*^15^N values are present in graminoids (grasses and sedges), herbs, and forbs than in shrubs and trees [21, 22]. It was this difference that made it possible to distinguish grazing and browsing herbivore species.

The application of analyses of carbon and nitrogen stable isotope composition in revealing past ecosystems requires a good referential data set of modern animals. Inferring differences in climatic and environmental conditions on the basis of animal bone collagen carbon and nitrogen values requires more detailed recognition of the factors influencing the isotopic composition of their tissues. This requires knowledge of the variability between individuals of the same species dwelling in various habitats. To our best knowledge, there are only a few studies comparing bone collagen isotopic compositions of modern free-ranging animals inhabiting various locations of well-known climatic conditions and habitats, and there are still many questions and hypotheses that need to be verified. Drucker et al. [15] reviewed in detail the *δ*^13^C values of modern large herbivores from open and closed environments. They linked the *δ*^13^C variability with the observed “canopy effect,” but other environmental factors, which potentially could have had an impact on the stable isotopic composition, were not considered. A detailed study on climatic and habitat factors influencing *δ*^13^C and *δ*^15^N values in bone collagen was conducted on white-tailed deer (*Odocoileus virginianus*), but the number of individuals analyzed in this study was relatively low [23]. A larger number of individuals were analyzed by Stevens et al. [2] in a comparative study of five red deer (*Cervus elaphus*) populations; however, they did not apply a correction for the *δ*^13^C shift in the atmospheric CO_2_ in their analyses. The rapid decrease in the *δ*^13^C value of atmospheric CO_2_ associated with the burning of fossil fuel and deforestation is passed on first to plants and then to herbivores. In order to compare carbon isotopic values of samples collected in different years, it is necessary to correct their *δ*^13^C values [see e.g., 24, 25 for more details]. The association between isotopic compositions and habitat in general is well-established; however, it is still unknown which of the climatic and environmental factors best explain the *δ*^13^C and *δ*^15^N values in the bone collagen of ungulates.

In our research, we used red deer as a model ungulate species, as they are widely distributed across Europe and show great ecological flexibility. Red deer inhabit a wide spectrum of habitats from dense forests to steppes [26], and their diet consists of different plant species, including trees, shrubs, grasses, sedges, and herbs [27]. In our analyses, we used a large number of samples (N = 242) from 15 study sites and rigorous statistical methods to indicate which of the four climatic and four environmental factors explain in the best way isotopic variability in the bone collagen of red deer individuals. The study sites represented various habitats (from open coastal to closed mountainous and densely forested areas) and were characterized by different climatic conditions. We predicted that the ranges of *δ*^13^C and *δ*^15^N values, corresponding to forested, mosaic and open areas, could be used as reference data to reveal the environments of occurrence of different ungulate species in palaeoecological studies. We hypothesized that in bone collagen of red deer (i) *δ*^13^C values would be negatively influenced by the proportion of forest cover and the annual precipitation, while the mean January temperature and the altitude would be positively associated with *δ*^13^C values; (ii) *δ*^15^N values would be positively influenced by the proportion of open area and the mean January temperature and negatively influenced by the annual precipitation and the altitude; (iii) *δ*^15^N and *δ*^13^C values would decrease with increasing distance to the seashore in coastal habitats (coastal areas); and (iv) red deer from homogenous habitats (densely forested or open) would show significantly lower variability of *δ*^15^N and *δ*^13^C values than individuals from mosaic areas.

## 2. Materials and methods

### 2.1. Study area

**Fig 1:**
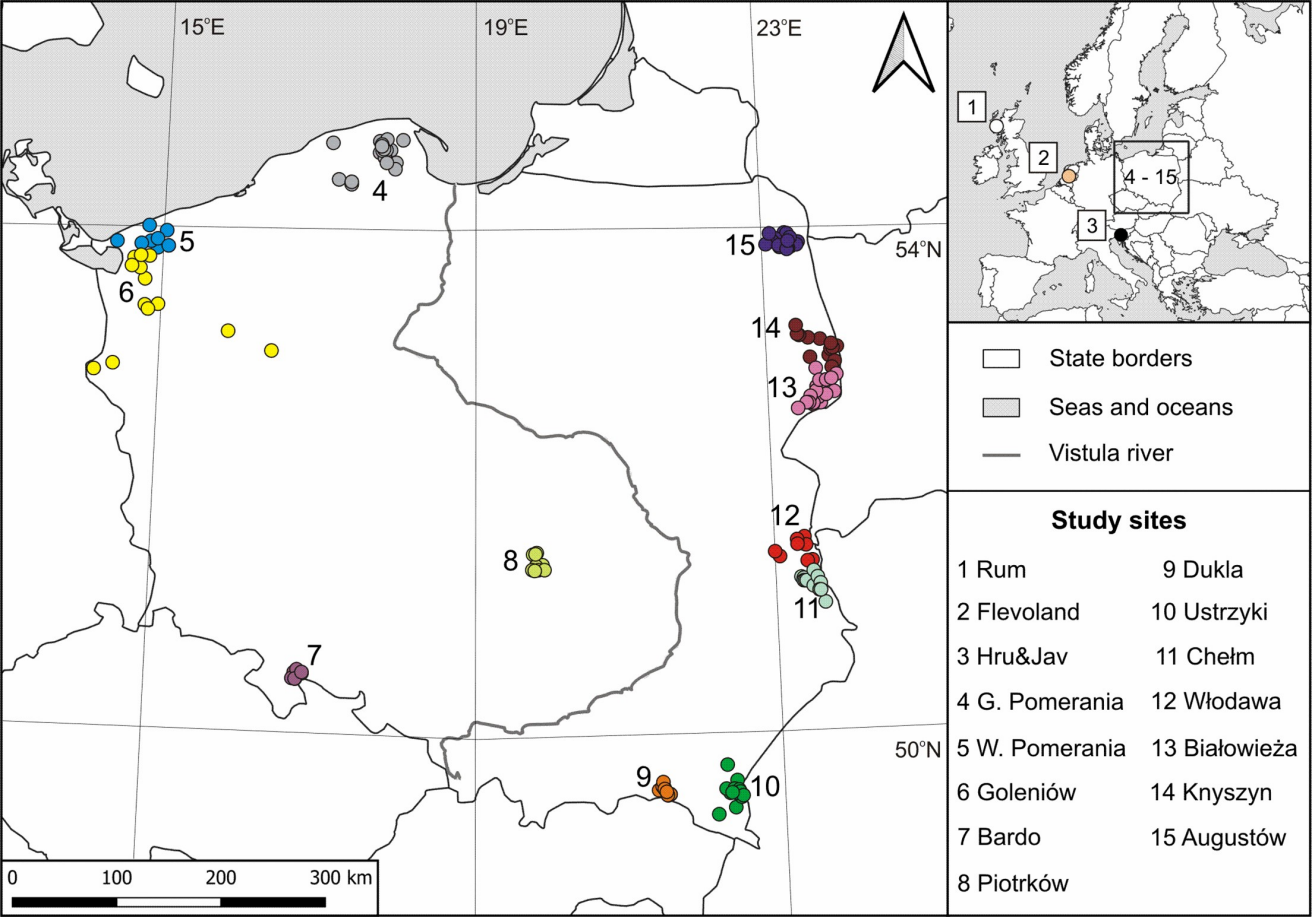
Map of geographical locations of the study sites. The map was created with the ArcGIS 10.3.1 (license no ESU 727916487) software based on a source map from the public Natural Earth website (http://www.naturalearthdata.com/).

Red deer bone samples (mandibles, finger bones, or metatarsus) for isotopic analyses were collected from 15 study sites, including 12 locations in Poland, one in Scotland (UK), one in Slovenia, and one in the Netherlands (Fig 1). The names of the study sites refer to geographic names of the areas (study sites from 1 to 5), forest districts (6 to 12), and woodlands (13 to 15) where they were collected. More details concerning the study sites (their full names, coordinates, habitats with dominant tree species, number of sampled individuals) can be found in S1 Table. The study area covered various habitats: (1) large woodlands (e.g., Białowieża Primeval Forest), (2) mosaic of meadows, arable grounds, and forest areas (e.g., Chełm), and (3) grasslands (e.g., the Isle of Rum). The sampled red deer were free-ranging individuals, except those from Flevoland (the Netherlands), which inhabited a fenced reserve covering ca. 56 km^2^.

The mean annual temperature in the study sites located in Poland ranged from 6.6°C (Ustrzyki and Knyszyn) to 8.6°C (Goleniów), while in Scotland (UK), Slovenia, and the Netherlands, the mean annual temperature amounted to 8.7°C, 8.4°C, and 9.6°C, respectively. In Poland, the highest annual precipitation was 835 mm (Ustrzyki) and the lowest was 529 mm (Włodawa). Annual precipitation at the study sites in Scotland (UK), Slovenia, and the Netherlands was 1928 mm, 1806 mm, and 781 mm, respectively. The sites are situated at different altitudes, from −3 m above sea level (a.s.l.) in Flevoland to 562 m a.s.l. in Hru&Jav (Slovenia). More details concerning the climatic conditions (mean annual temperature, mean July temperature, mean January temperature, annual precipitation) and the altitude of all study sites are presented in S2 Table.

In all study sites in Poland and Slovenia, game animals (including red deer) were supplementarily fed mainly for harvest purposes. Additionally, they were fed during the winter time (more intensively in severe and snowy than in mild winters) and potentially had access to agricultural crops located near forest areas. However, it was impossible to assess the amount of supplementary food or crops consumed by each of the studied individuals, and there were also no data available concerning the amount of supplementary food provided in each of the study sites. Red deer rarely caused any damage to agricultural crops, and such losses were not recorded. Additionally, there are no formal recommendations concerning the amount of supplementary food that should be provided for wild ungulates in Poland. Such food usually is not isotopically homogenous as it may consist of hay, straw, dried shoots with leaves as well potatoes, beets, carrots, cabbage, silage, and cereal grains (e.g., corn [*Zea mays*]). Therefore, we were not able to include supplementary feeding or crop availability as variables in our analyses. Seasonally, in study sites located in uplands and mountains (Hru&Jav, Bardo, Ustrzyki, Dukla), red deer migrated between lower and higher altitudes.

### 2.2. Sample collection

Red deer bone samples were collected from 1965 to 2019 and stored in the zoological collection of the Mammal Research Institute PAS in Białowieża, Poland. They came from legally harvested individuals and were provided by hunters belonging to the Polish Hunting Association, foresters of the Polish State Forests Holding, game trading companies in Poland, members of the Hunters’ Association of Slovenia, and workers of Staatsbosbeheer in the Netherlands. Samples from the Isle of Rum in Scotland were collected by a field assistant from the Isle of Rum Red Deer Project and came from red deer that had died of natural causes and were found in the field. None of the animals were killed intentionally for this study; therefore, no permission was required for the collection of any of these samples.

### 2.3. Sample preparation and analysis

Before analyses, the collected bone samples were frozen at −20°C. Prior to collagen extraction, the samples were rinsed in water and dried at 70°C for 6 hours. Bone collagen was extracted using the classical Longin method [28] with additional treatment as described by O’Connell et al. [29]. The bone samples were crushed into chunks and sieved through a 2-mm mesh. About 300 mg of chunks were defatted by soaking in (1) NaOH [30] or (2) dichloromethane and methanol (2:1 v/v) [31]. For details concerning methods of defatting and a comparison of the results obtained, see Sykut et al. [32]. After defatting, the samples were demineralized in 6 ml of 0.5 M hydrochloric acid for 24 h with one solvent change until complete decalcification. Then, they were gelatinized by heating in pH 3.0 aq. HCl at 80°C for 12 h, centrifuged for 5 min (4000 r/min), and dried at 80°C for 3 days. In the next step, the dried collagen was weighed into tin capsules. Three subsamples of each collagen sample were prepared for the measurements. The elemental and isotopic measurements were performed in the Laboratory of 14C and mass spectrometry, Silesian University of Technology (Gliwice, Poland) using an IsoPrime EA-CF-IRMS continuous flow isotope ratio mass spectrometer connected to the EuroVector elemental analyzer. The obtained carbon and nitrogen isotope measurements were calibrated to VPDB and AIR standards, respectively [33, 34]. The stable isotope values were expressed in isotope delta (*δ*) notation as follows:

$$\delta^{13}C=\left[\frac{({}^{13}C/{}^{12}C){}_{sample}-({}^{13}C/{}^{12}C){}_{{}_{VPDB}}}{({}^{13}C/{}^{12}C){}_{VPDB}}\right]*1000(‰)$$

and

$$\delta^{15}N=\left[\frac{({}^{15}N/{}^{14}N){}_{sample}-({}^{15}N/{}^{14}N){}_{{}_{AIR}}}{({}^{15}N/{}^{14}N){}_{AIR}}\right]*1000(‰)$$

The *δ*^13^C and *δ*^15^N values are presented in units of parts per thousand and communicated in per mil shown as ‰ [35]. Samples of collagen were routinely calibrated to international standards. The *δ*^13^C values were calibrated to values of IAEA–C8 (*δ*^13^C = −18.31‰) and IAEA–C5 (*δ*^13^C = −25.49‰). The *δ*^15^N values were calibrated to values of IAEA–NO_3_ (*δ*^15^N = 4.7‰) and IAEA–USGS34 (*δ*^15^N = −1.8‰). C/N elemental ratio values were calibrated to values of Aspartic Acid (elemental composition: C– 36.09%, H– 5.30%, N– 10.52%, O– 48.08%) and UREA (elemental composition: C– 20%, H– 6.71%, N– 46.65%, O– 26.64%). The precision of these methods is 0.1‰ for *δ*^13^C and 0.2‰ for *δ*^15^N.

The analyzed samples originated from deer that died in different years, and the *δ*^13^C composition of global atmospheric CO_2_ varies over time [36]. Due to anthropogenic CO_2_ emissions, the *δ*^13^C values measured in the collagen have been corrected for the shift in *δ*^13^C values according to following formula: *δ*^13^C_atm_ = −6.429 ± 0.0060 exp [0.0217(*t −* 1740)], where *δ*^13^C_atm_ is the correction added to the *δ*^13^C value and *t* is the year of the individual’s death [24]. Uncorrected values of *δ*^13^C, atmospheric correction values and corrected values of *δ*^13^C are provided in S3 Table.

### 2.4. Spatial and climatic data

We georeferenced the locations of red deer samples (where the animals were culled or found dead). When precise data were not available, we assumed the centroid of the hunting district, where the animals were culled or found dead, to be the sample location. For each location, we described the following environmental variables: (1) the proportion of forest cover (later called forest cover), (2) the proportion of open area (later called open area), (3) the mean annual temperature, (4) the mean January temperature, (5) the mean July temperature, (6) the annual precipitation, and (7) the altitude. Additionally, for individuals from study sites situated in coastal areas, we measured the distance to the seashore or coastal salt lake (later referred to as distance to the seashore).

The proportion of forest cover and open area at the sample locations were estimated within circular buffers of 3 km and 5 km radii for females and males, respectively. The buffer size corresponded to the mean annual home range size of does and stags of red deer in Europe [37]. The results of our previous study showed that generally there were no differences in isotopic composition between male and female red deer [32], so we did not include sex as variable in our analyses. The distance to the seashore and the proportion of forest cover and open area were estimated from CORINE Land Cover maps [38] using ArcGIS 10.3.1 [39] software. Climatic data (mean annual temperature, mean January temperature, mean July temperature, annual precipitation) were obtained from WorldClim Ver. 2 data sets [40], providing data at the spatial resolution of 30 arc-seconds (~1 km). Elevation data were downloaded from the EROS Center [41].

### 2.5. Statistical analyses

We tested the correlation between the values of *δ*^13^C and *δ*^15^N for all analyzed samples with Pearson’s correlation analysis. We calculated the mean ± SE (standard error of the mean) of *δ*^13^C and *δ*^15^N values for red deer inhabiting each of the study sites (S2 Table). To test the differences for significance in *δ*^13^C and *δ*^15^N values among individuals from different study sites, we used Kruskal–Wallis analysis of variance. These analyses were performed in Statistica ver. 7 [42].

We used linear mixed-effects models (LMMs) with a Gaussian error structure to test for associations between stable isotope composition (*δ*^13^C or *δ*^15^N) and the following variables: percentage of forest cover, percentage of open area, mean January temperature, annual precipitation, and altitude. As we sampled red deer from the same locations multiple times, we set location and year as random factors in all our LMMs. The homoscedasticity in distribution of final model residuals was checked by visual inspection of plots presenting model residuals against fitted values (estimated responses). We ran separate models with *δ*^13^C and *δ*^15^N values as the response variables. All LMM models were performed using the *lme4* package [43] implemented in R version 4.0.2 [44].

To test which set of variables best explained the observed variance in *δ*^13^C and *δ*^15^N values, we created two sets (one for *δ*^13^C and one for *δ*^15^N) of competing models. Each model consisted of the uncorrelated independent variables (predictors). We assumed that the predictors were uncorrelated when Pearson’s correlation coefficients were below |0.6| (S4 Table). Next, the competing models were ranked according to Akaike’s Information Criterion (AIC) with the second-order correction for a small sample size (AICc) [45] using the *MuMin* package [46] implemented in R version 4.0.2 [44]. All models close to the top model (lowest AIC_c_) and with ΔAIC < 2 were considered to have substantial empirical support.

Since the coastal habitats could represent enriched carbon nitrogen stable isotope values [17], we tested the association between *δ*^13^C or *δ*^15^N values and the distance to the seashore. We performed additional LMMs for *δ*^13^C and *δ*^15^N values on selected samples (N = 61) of red deer individuals inhabiting areas at a distance ≤ 40 km from the seashore in the Rum, Flevoland, G. Pomerania, W. Pomerania, and Goleniów study sites.

To check for significant differences in the variability of *δ*^13^C or *δ*^15^N values between red deer occurring in habitats differing in forest cover, we assigned sampled individuals to four forest cover classes (0–20%, 21–50%, 51–80%, and 81–100% forest cover) and compared the variance of *δ*^13^C or *δ*^15^N values among those forest cover classes with ANOVA fitted on residual values and Tukey’s post hoc HSD test. These analyses were completed in R version 4.0.2 [44].

## 3. Results

### 3.1. Variability of *δ*^13^C and *δ*^15^N in red deer from different study sites

**Fig 2:**
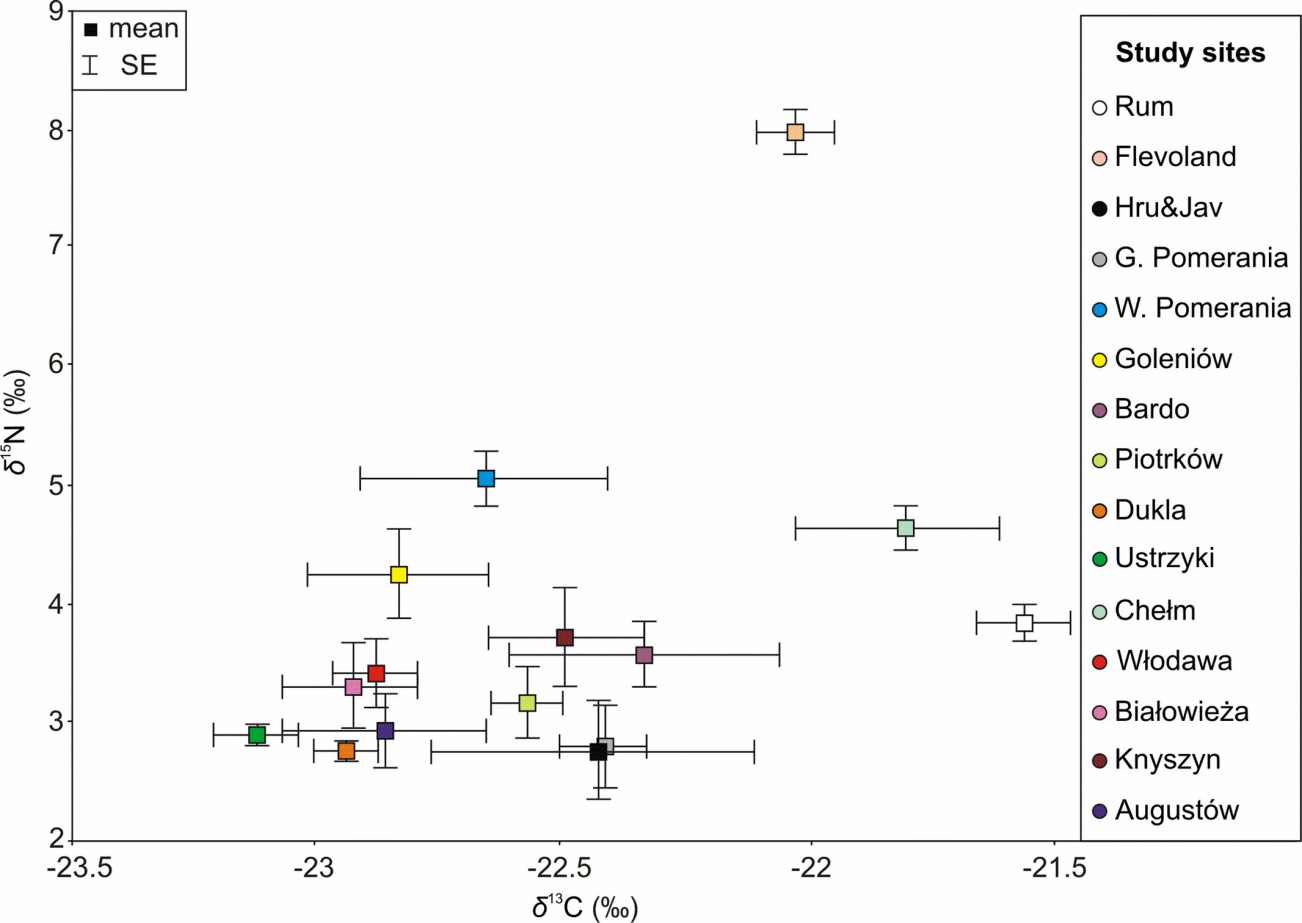
Mean values and standard errors of the mean (SE) of stable carbon and nitrogen stable isotope compositions (*δ*^13^C and *δ*^15^N values) in bone collagen of red deer inhabiting different study sites.

The values of *δ*^13^C ranged from −24.83 to −19.50‰, and those of *δ*^15^N from 0.60 to 9.29‰, respectively. Values of *δ*^13^C and *δ*^15^N were significantly correlated (r = 0.42, N = 242, *P* < 0.001, S1 Fig). Ranges of *δ*^13^C and *δ*^15^N values of individuals from different study sites overlapped (S1 Fig). The highest mean *δ*^13^C value (−21.55 ± 0.09‰) was detected in individuals from the Rum covered mostly by heathlands, while the lowest mean value (−23.13 ± 0.08‰) was observed in red deer from the Ustrzyki study site–a mountainous area covered by forests (Fig 2, S2 Table). The highest mean *δ*^15^N value (7.89 ± 0.19‰) was observed in deer from the Flevoland–a fenced wetland covered mostly by grasslands, and the lowest mean value of *δ*^15^N (2.76 ± 0.42‰) was detected in bone collagen of deer from Hru&Jav–a mountainous area covered mostly by forests (Fig 2, S2 Table). Pairwise comparisons showed significant differences in *δ*^13^C in 14 out of 105 pairs of study sites and in *δ*^15^N in 18 out of 105 pairs (S5 Table). The most different values of *δ*^13^C from the studied red deer were found among individuals inhabiting the Isle of Rum, which differed significantly in 6 out of 14 study sites comparisons. The most different values of *δ*^15^N from the studied red deer were found in specimens from Flevoland, which differed significantly in 10 out of 14 study sites comparisons (S5 Table).

### 3.2. Environmental factors best explaining the variability of *δ*^13^C and *δ*^15^N in red deer inhabiting different habitats

**Fig 3:**
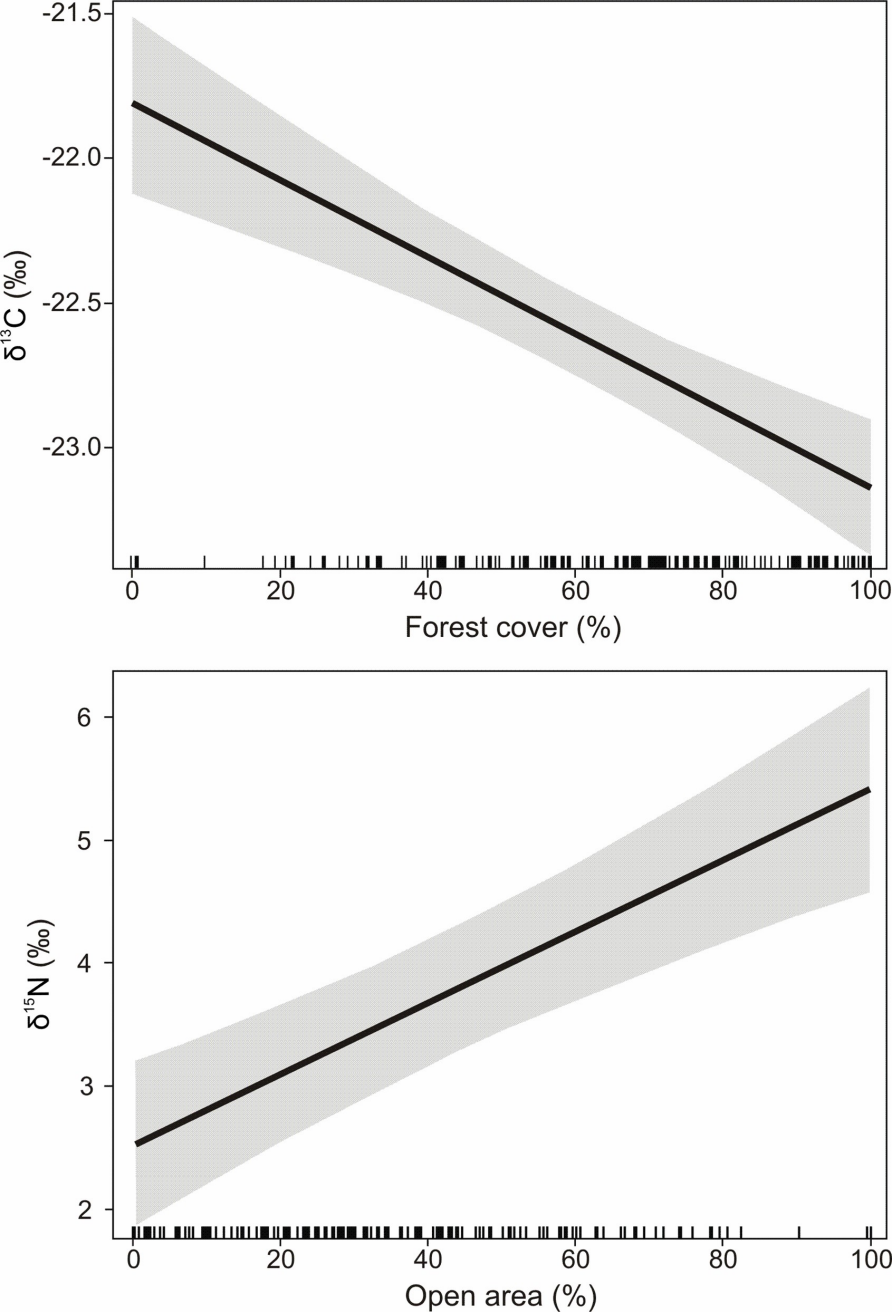
Influence of forest cover (top) and open area (bottom) in buffers around red deer localities on carbon and nitrogen stable isotope compositions (*δ*^13^C and *δ*^15^N values) in bone collagen of the studied individuals based on estimates from multiple regression models. Grey areas– 95% confidence intervals of the regression lines.

Based on the AICc criteria, the best model explaining the variation in *δ*^13^C values in bone collagen of red deer was the top-ranked model, which consisted of a single variable–percentage of forest cover (Table 1). The other models had ΔAICc < 2 and were therefore discounted. Percentage of forest cover was negatively associated with *δ*^13^C values (slope = −1.40 ± 0.20, *t* = −6.92, *P* < 0.001). With the increasing percentage of forest cover from 0 to 100%, *δ*^13^C values decreased from −21.75‰ to −23.15‰ (Fig 3, top).

**Table 1:** Model selection (based on the AICc criteria) of considered multiple regression models aiming to investigate the effect of forest cover, mean January temperature, annual precipitation, and altitude on *δ*^13^C values in bone collagen of red deer from different sampling sites in Europe.

Parameters included	df	AICc	ΔAICc	*ωi*
Forest cover + Year + Site ID	5	494.9	0.00	0.9995
Altitude + Forest cover + Year + Site ID	6	511.1	16.2	0.0003
Forest cover + Annual precipitation + Year + Site ID	6	511.9	17.1	0.0002
January mean temperature + Year + Site ID	5	524.1	29.3	0.0000
Intercept + Year + Site ID	4	526.4	31.5	0.0000
Altitude + Forest cover + Annual precipitation + Year + Site ID	7	527.9	33.0	0.0000
Altitude + January mean temperature + Year + Site ID	6	538.9	44.0	0.0000
January mean temperature + Annual precipitation + Year + Site ID	6	540.8	45.9	0.0000
Altitude + January mean temperature + Annual precipitation + Year + Site ID	7	554.6	59.7	0.0000

Site ID and sampling year were random factors. The top-ranked model representing the highest parsimony (the lowest AICc scores) was selected as the best model; df—number of estimated parameters; AIC_c_—Akaike’s information criterion with a second order correction for small sample sizes; ΔAIC_c_—difference in AIC_c_ between a given model and the most parsimonious model; *ωi—*weight of the model.

The best model explaining the variation in *δ*^15^N values was the top-ranked model, which included the percentage of open area as a single explanatory variable (Table 2). The other models had ΔAICc < 2 and were therefore discounted. The percentage of open area was positively related to the *δ*^15^N value (slope = 2.76 ± 0.50, *t* = 5.55, *P* < 0.001). The selected model predicted a 2.76‰ increase in the *δ*^15^N value in bone collagen between red deer inhabiting 0 and 100% open area (Fig 3, bottom).

**Table 2:** Model selection (based on the AICc criteria) of considered multiple regression models aiming to investigate the effect of forest cover, mean January temperature, annual precipitation, and altitude on *δ*^15^N values in bone collagen of red deer from different sampling sites in Europe.

Parameters included	df	AICc	ΔAICc	ωi
Open area + Year + Site ID	5	814.1	0.00	0.9946
Open area + Annual precipitation + Year + Site ID	6	825.8	11.6	0.0030
Altitude + Open area + Year + Site ID	6	826.2	12.0	0.0025
Intercept + Year + Site ID	4	836.3	22.2	0.0000
January mean temperature + Year + Site ID	5	836.5	22.3	0.0000
January mean temperature + Annual precipitation + Year + Site ID	6	843.5	29.4	0.0000
Altitude + January mean temperature + Year + Site ID	6	844.3	30.2	0.0000
Altitude + Open area + Annual precipitation + Year + Site ID	7	839.3	25.0	0.0000
Altitude + January mean temperature + Annual precipitation + Year + Site ID	7	855.9	41.7	0.0000

Site ID and sampling year were random factors. The top-ranked model representing the highest parsimony (the lowest AICc scores) was selected as the best model; df -number of estimated parameters; AIC_c_—Akaike’s information criterion with a second order correction for small sample sizes; ΔAIC_c_—difference in AIC_c_ between a given model and the most parsimonious model; *ωi—*weight of the model.

**Fig 4:**
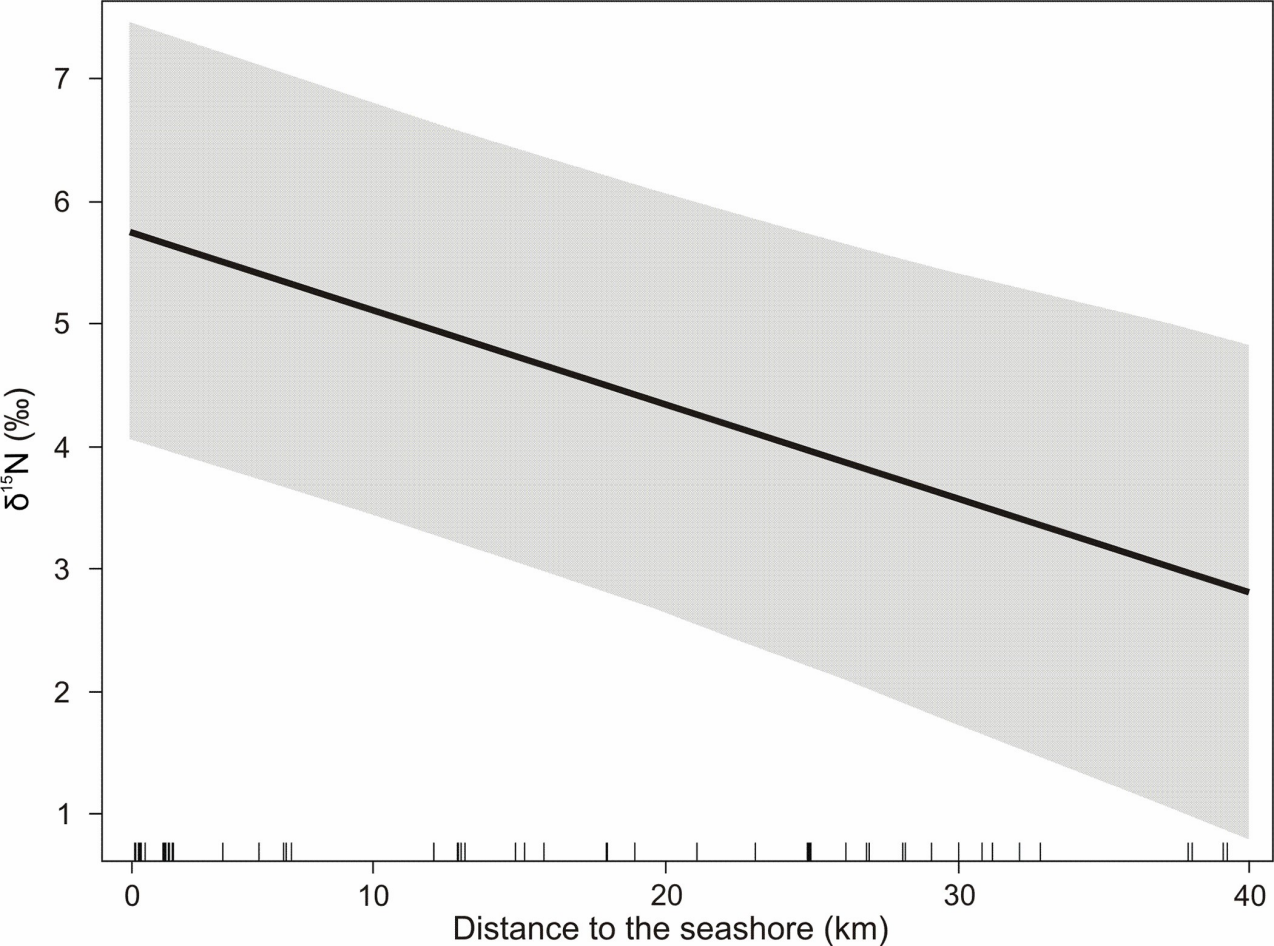
Relationship between distance to the seashore and *δ*^15^N values of bone collagen of red deer inhabiting coastal sampling sites located not more than 40 km from the sea. Grey area– 95% confidence interval of the regression line.

For red deer inhabiting study sites located in coastal areas (<40 km from the seashore), distance to the seashore did not explain the variation in *δ*^13^C (slope = −0.01 ± 0.01, *t* = −0.92, *P* = 0.36). In the case of the nitrogen model, distance to the seashore had a significant effect on *δ*^15^N values (slope = −0.08 ± 0.02, *t* = −3.92, *P* < 0.001). With increasing distance to the seashore from 0 to 40 km, *δ*^15^N values decreased from 5.91‰ to 2.82‰ (Fig 4).

### 3.3. Differences in the variability of *δ*^13^C and *δ*^15^N values among red deer groups inhabiting open, mosaic, and forested areas

**Fig 5:**
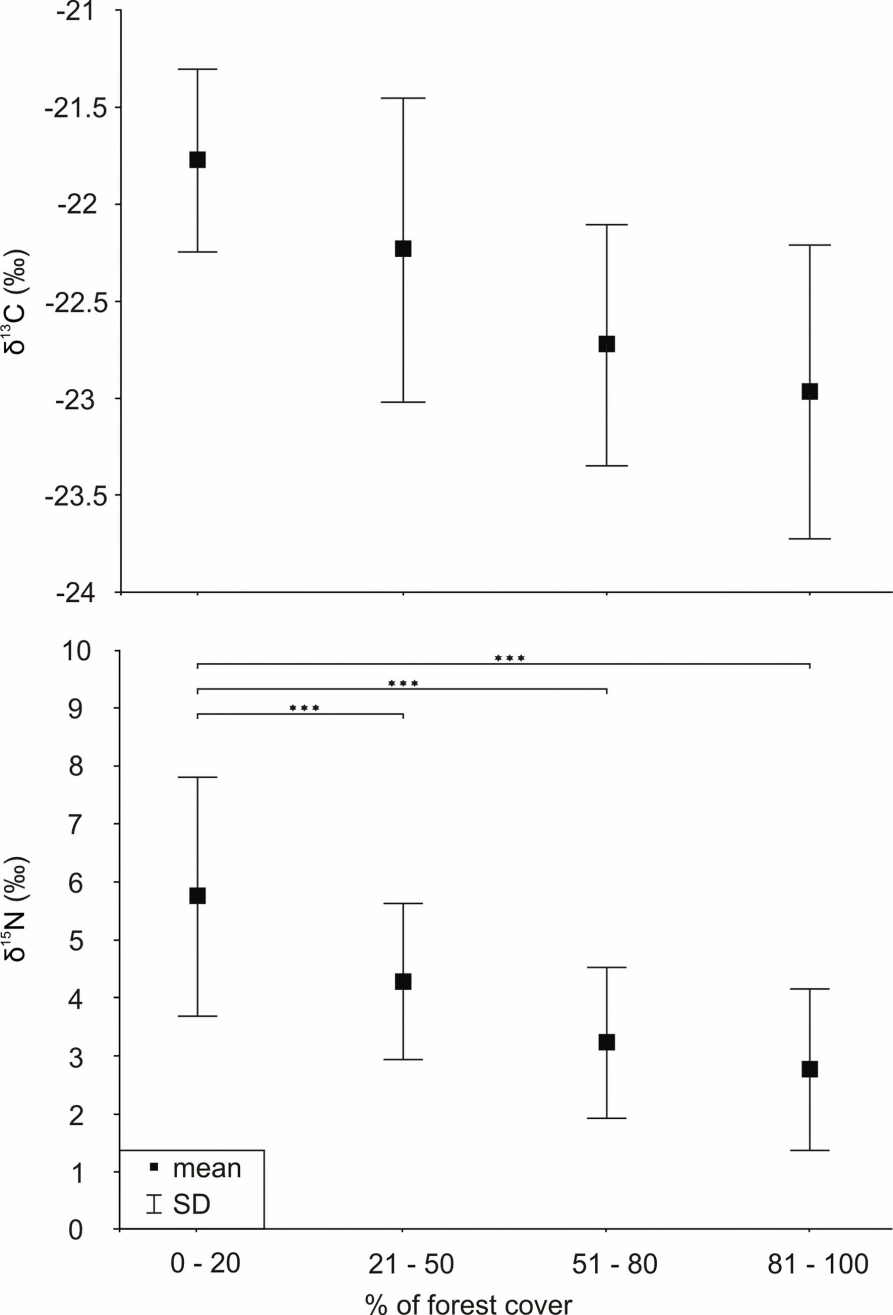
Mean values and standard deviation (SD) of *δ*^13^C (top) and *δ*^15^N (bottom) in red deer bone collagen grouped by percentage of forest cover in buffers around the localities of the studied individuals. Statistical significance of the variance by Tukey’s HSD test: ***P < 0.001.

As the percentage of forest cover and percentage of open area summed to 100%, and these parameters best explained the variability of *δ*^13^C and *δ*^15^N values, respectively (Fig 3), we divided the studied individuals into four groups according to the percentage of forest cover in their buffers. To evaluate whether the variability in stable isotope composition of carbon and nitrogen differed between red deer individuals inhabiting various habitats (open, mosaic, or forested areas), we compared standard deviations of *δ*^13^C and *δ*^15^N values among groups of analyzed specimens, determined according to the percentage of forest cover (0–20%, 21–50%, 51–80%, and 81–100%) in red deer buffers (Fig 5). The lowest variability in *δ*^13^C was detected in the group with the lowest forest cover (SD = 0.47), and the highest in groups with 21–50% (SD = 0.78) and 81–100% (SD = 0.76) forest cover. However, the differences in variability of carbon isotopic composition between all pairs of groups were not significant (*P* > 0.05). The highest variability of nitrogen isotopic composition were observed in the group with 0–20% of forest cover (SD = 2.05) and the lowest in the groups with 21–50% and 81–100% forest cover (SD = 1.35 and SD = 1.39, respectively). The difference in variability of *δ*^15^N was significant only between the first (0–20% forest cover) and the three other groups (*P* < 0.001) (Fig 5).

## 4. Discussion

Among all analyzed climatic and environmental factors, the percentage of forest cover best explained the variability of *δ*^13^C values in the bone collagen of red deer. Individuals from forested areas, dwelling under the canopy, showed lower *δ*^13^C values than deer from more open areas. These results are concordant with the results of previous stable isotopic studies, including those one modern free-ranging cervids and bovines [15, 47]. The canopy cover effect results from depletion of ^13^C abundance in plants growing under a closed canopy, in poorly ventilated, more humid and shaded conditions, compared to those from open habitats [14, 48]. Since herbivores incorporate the isotopic signatures of the food they consume into their tissues, the difference between plants from open and closed environments was reflected in the *δ*^13^C values of red deer bone collagen analyzed in this study.

The general pattern of nitrogen stable isotope composition in bone collagen presented in this study was as follows: deer from open lands showed higher *δ*^15^N values than deer inhabiting forested areas. Similar results were reported in the study on the European bison (*Bison bonasus*). The variability of *δ*^15^N in bone collagen of European bison was negatively influenced by forest cover and positively associated with the average annual temperature and the use of agricultural crops [47]. Also, in a study on South American herbivores, huemul (*Hippocamelus bisulcus*) inhabiting the Patagonian Andean forest presented lower *δ*^15^N values than the guanaco (*Lama guanicoe*) occupying Patagonia’s continental steppe [49]. The authors explained their results as the effect of a difference in precipitation level among the studied habitats. Similar negative correlations between the quantity of precipitation and modern faunal *δ*^15^N values were reported in a study performed in South Africa [50], but this relationship was observed in areas with rainfall lower than 400 mm per year. The annual precipitation in all sites in the present study was higher than 500 mm per year. In our study, the proportion of open area was the most significant factor influencing *δ*^15^N values, while other variables such as altitude, annual precipitation, or mean January temperature were not significant.

Due to the presumed enrichment in ^15^N in red deer from coastal areas relative to those from inland areas, we tested the hypothesis that distance to the seashore significantly influenced the nitrogen isotopic composition of bone collagen. Bone collagen *δ*^15^N values of deer living in coastal areas (<40 km from the seashore) decreased significantly with increasing distance from the seashore. A possible explanation may be given by the sea spray effect or soil salinity. Sea spray contains marine nitrogen, which affects forage in coastal areas. Such nitrogen is enriched in ^15^N in comparison to terrestrial nitrogen and is deposited near the seashore and taken up by plants [51]. Soil salinity contributes to ^15^N enrichment in plants from such saline areas and is later recorded in the herbivores which consume them [52]. The sea spray effect on vegetation adapted to highly saline coastal substrates is related to the high *δ*^15^N values also in Pampas Deer (*Ozotoceros bezoarticus*) in Eastern Central Argentina [53].

In general, carbon isotopic values were highly variable among red deer from a single study site and within groups determined by the percentage of forest cover. There are several potential explanations for such variability. Firstly, regardless of whether red deer inhabited the same study site, they could have fed in areas with different percentages of forest cover, as most of our studied sites were not homogenous according to the available habitats. Moreover, the proportion of forest cover does not always reflect the closeness of the canopy and condition of the understory, which may determine the “canopy cover effect”. For instance, in managed forests, where clear-cuts are practiced, ^13^C depletion may not be present [e.g., 54]. Secondly, due to the fact that red deer is one of the most important game species across Europe, individuals from all study sites, except Rum and Flevoland, were affected by anthropogenic interference, i.e., foraging on supplementary food or cultivated crops. Since the supplementary food or agricultural crops may contain components with higher *δ*^13^C values than native plants (e.g., corn [55]), the carbon isotopic composition of bone collagen may have artificially increased values. Similarly, hay mowed in open areas and delivered to the forest would attenuate the isotopic signals of forest habitats [56]. Despite the fact that the red deer analyzed in present study were supplementarily fed and had an access to crops, they presented the same pattern of *δ*^13^C values as caribou that were not supplementarily fed and had no access to crops. Hair of modern caribou from closed canopy habitats (i.e., boreal forest) were depleted in ^13^C relative to hair of individuals from open environments (i.e., tundra) [15, 57]. Finally, the variability within study site may also be shaped by individual food preferences [58]; therefore, the effect of supplementary feeding may differ between individuals from a single study site.

Similar to *δ*^13^C, *δ*^15^N values were highly variable among red deer from a single study site, and within groups determined by the percentage of forest cover. Again, there are a number of potential explanations for this variability. Firstly, we cannot exclude the possibility that the sampled individuals (except red deer from the Rum and Flevoland study sites) fed on agricultural crops or supplementary food provided by hunters or foresters. The consumption of cultivated plants by red deer (as in the case of *δ*^13^C) increased the *δ*^15^N values in their tissues [59]. Secondly, due to the fact that, in large herbivores, *δ*^15^N values in hair increase during nutritional stress [60], it is possible to influence their bone collagen values as well. This may explain the high *δ*^15^N values in the Flevoland deer, which were in poor condition due to starvation [61]. On the other hand, in open areas with low habitat productivity, e.g., as in our study site in the Isle of Rum [62], the *δ*^15^N abundance can be relatively low in the soil and plants [63] and, in consequence (as reflected in our study), in the bone collagen of red deer. Thus, low productivity of habitats of occurrence can also increase the variance in nitrogen isotopic values in the bone collagen of studied herbivores. Finally, as in the case of carbon isotopic values, we cannot exclude interindividual variability of food preferences among individuals from the same study site [58].

## 5. Conclusions

On the whole, the results of our study confirmed that the carbon and nitrogen stable isotope compositions of red deer bone collagen across a wide range of environments are shaped by habitat conditions. Using rigorous statistical methods, we identified forest cover (or alternatively, open area) as the factor that best described the isotopic composition of carbon and nitrogen in the bone collagen of modern red deer in Europe. The association of forest cover and *δ*^13^C of red deer confirmed the presence of the “canopy cover effect.” The variability of *δ*^15^N values was best explained by the proportion of open area. Additionally, in coastal areas located less than 40 km from the shore, the variability of *δ*^15^N values was influenced by the marine environment. However, the variance of the carbon and nitrogen isotopic composition of bone collagen was high among individuals inhabiting similar environmental conditions, so it can be concluded that they were influenced by additional factors, which were not identified and analyzed in our study.

In the context of palaeoecological studies, we highly recommend the use of additional sources of information about past habitats of studied herbivore species. e.g., palynological data, for more accurate interpretation of the results of stable isotopic analyses. Despite this high variation in *δ*^15^N and *δ*^13^C values, the present large-scale study showed a general association between bone collagen isotopic composition and the habitat.

## Supporting information

S1 Table Characteristics of the study sites. (DOCX)

S2 Table Mean (± SE) values of *δ*^13^C and *δ*^15^N (‰) in bone collagen of red deer and characteristics of climatic conditions at the study sites where the analyzed samples were collected. (DOCX)

S3 Table Climatic, environmental, and stable isotope data used in the study. (DOCX)

S4 Table Pairwise correlation matrix of selected climatic and environmental parameters of red deer from all sampling sites. (DOCX)

S5 Table Statistical differences between *δ*^13^C and *δ*^15^N values in bone collagen of red deer inhabiting different study sites. (DOCX)

S1 Fig Stable carbon (*δ*^13^C) and nitrogen (*δ*^15^N) isotope signatures of red deer from different study sites. (TIF)
