# Supplementary material for: Environmental factors shaping stable isotope signatures of modern red deer (Cervus elaphus) inhabiting various habitats
Source: PLoS One. 2021 Aug 13;16(8):e0255398. doi: 10.1371/journal.pone.0255398 (PMC8362983; doi:10.1371/journal.pone.0255398)
Supplement: S1 Table — (DOCX) [file pone.0255398.s001.docx]

**Environmental factors shaping stable isotope signatures of modern red deer (*Cervus elaphus)* inhabiting various habitats**

Maciej Sykut*, Sławomira Pawełczyk, Tomasz Borowik, Boštjan Pokorny, Katarina Flajšman, Tjibbe Hunink, Magdalena Niedziałkowska

Corresponding author: Maciej Sykut mail: msykut@ibs.bialowieza.pl

S1 Table. Characteristics of the study sites (data from web page: <https://www.bdl.lasy.gov.pl/portal>, access 1 July 2020;[1-3])

| Site No | Full name of the study site, country abbreviation | Short name of the study site | Coordinates | Habitat | Mean % of forest cover±SE | Dominant tree species | Number of individuals (male to female ratio) |
| --- | --- | --- | --- | --- | --- | --- | --- |
| 1 | Isle of Rum, Scotland, UK | Rum | 6°27–6°14’ W, 57°03–56°56’N | Grasslands, heathland, bogs and fens, heathers | 0.34 ± 0.11 | – | 10 (5:5) |
| 2 | Oostvaardersplassen reserve, Province of Flevoland, NL | Flevoland | 5°19–5°24’ E,  52°26–52°28’N | Grasslands, reed vegetation, semi-open mosaic vegetation of reed, tall herbs and trees | 0.09 ± 0.00 | – | 11 (0:11) |
| 3 | Hrušica and Javorniki Hills, SI | Hru&Jav | 13°58–14°19’E, 45°58–45°43’N | Mixed forests | 66.91 ± 6.80 | Silver fir (*Abies alba*), European beech (*Fagus sylvatica*) | 12 (6:6) |
| 4 | Gdańsk Pomerania, PL | G. Pomerania | 17°30–18°11’E, 54°40–54°19’N | Mosaic of the mixed forests, reeds and shrubs | 53.58 ± 2.90 | Scots pine (*Pinus sylvestris*), European beech | 23 (4:19) |
| 5 | Western Pomerania, PL | W. Pomerania | 14°35–15°10’E, 54°01–53°53’N | Mosaic of the mixed forests and arable grounds | 35.08 ± 3.30 | Scots pine | 8 (0:8) |
| 6 | Goleniów Forest District, PL | Goleniów | 14°13–16°47’E, 54°1–52°51’N | Coniferous forests | 69.00 ± 7.00 | Scots pine | 13 (0:13) |
| 7 | Bardo Śląskie Forest District, PL | Bardo | 16°47–16°56’E 50°29–50°25’N | Coniferous and mixed forest | 56.29 ± 5.08 | Scots pine, English oak (*Quercus robur*), European beech, Norway spruce (*Picea abies*) | 16 (5:11) |
| 8 | Piorków Forest District, PL | Piorków | 19°45–19°55’E, 51°27–51°17’N | Coniferous forest | 64.53 ± 3.30 | Scots pine | 14 (4:10) |
| 9 | Dukla Forest District, PL | Dukla | 21°37–21°45’E, 49°31–49°26’N | Coniferous and mixed forest | 69.99 ± 1.61 | European beech, Silver fir | 22 (11 :11) |
| 10 | Ustrzyki Dolne Forest District, PL | Ustrzyki | 22°27–22°40’E, 49°40–49°20’N | Deciduous, mixed,  coniferous forests, meadows | 67.66 ± 1.72 | European beech, Silver fir | 22 (10:12) |
| 11 | Chełm Forest District, PL | Chełm | 23°01–23°41’E, 51°18–51°04’N | Mosaic of the mixed forest, meadows and arable grounds | 34.39 ± 2.67 | English oak, Scots pine, Silver birch (*Betula pendula*) Black alder (*Alnus glutinos*) | 18 (12:6) |
| 12 | Włodawa Forest District, PL | Włodawa | 23°07–23°35’E, 51°32–51°23’N | Coniferous forest | 65.80 ± 4.36 | Scots pine, Silver birch | 11 (7:4) |
| 13 | Białowieża Forest, PL | Białowieża | 23°27–23°53’E, 52°54–52°36’N | Deciduous and mixed forests | 89.77 ± 2.16 | Scots pine, Norway spruce, Black alder, English oak | 26 (10:16) |
| 14 | Knyszyn Forest, PL | Knyszyn | 23°14–23°53’E, 53°20–52°56’N | Coniferous forests | 64.86 ± 3.11 | Scots pine | 22 (11:11) |
| 15 | Augustów Forest, PL | Augustów | 22°58–23°22’E, 53°59–53°51’N | Coniferous forests | 91.34 ± 1.56 | Scots pine | 16 (16:0) |

**References**

1. Cornelissen P, Bokdam J, Sykora K, Berendse F. Effects of large herbivores on wood pasture dynamics in a European wetland system. Basic and Applied Ecology. 2014 2014/08/01/;15(5):396-406.

2. Klopcic M, Jerina K, Boncina A. Long-term changes of structure and tree species composition in Dinaric uneven-aged forests: are red deer an important factor? European Journal of Forest Research. 2010 2010/05/01;129(3):277-88.

3. Stevens R, Lister A, Hedges R. Predicting diet, trophic level and palaeoecology from bone stable isotope analysis: A comparative study of five red deer populations. Oecologia. 2006;149:12-21.
