# Supplementary material for: Environmental factors shaping stable isotope signatures of modern red deer (Cervus elaphus) inhabiting various habitats
Source: PLoS One. 2021 Aug 13;16(8):e0255398. doi: 10.1371/journal.pone.0255398 (PMC8362983; doi:10.1371/journal.pone.0255398)
Supplement: S2 Table — (DOCX) [file pone.0255398.s002.docx]

**Environmental factors shaping stable isotope signatures of modern red deer (*Cervus elaphus)* inhabiting various habitats**

Maciej Sykut*, Sławomira Pawełczyk, Tomasz Borowik, Boštjan Pokorny, Katarina Flajšman, Tjibbe Hunink, Magdalena Niedziałkowska

Corresponding author: Maciej Sykut mail: msykut@ibs.bialowieza.pl

S2 Table. Mean (± SE) values of *δ*^13^C and *δ*^15^N (‰) in bone collagen of red deer and characteristics of climatic conditions (ranges of: the mean annual temperature, the mean July temperature, the mean January temperature, the altitude, the annual precipitation) of sites from which red deer samples were collected.

| Site no | Short name of the study site | Mean *δ*^13^C  ± SE | Range | Mean *δ*^15^N  ± SE | Range | Annual mean temperature (°C) | July mean temperature (°C) | January mean temperature (°C) | Altitude  (m a.s.l.) | Annual precipitation (mm) |
| --- | --- | --- | --- | --- | --- | --- | --- | --- | --- | --- |
| 1 | Rum | -21.55 ± 0.09 | 0.98 | 3.84 ± 0.16 | 1.85 | 8.7 | 13.6 | 4.5 | 223 | 1928 |
| 2 | Flevoland | -22.03 ± 0.08 | 0.82 | 7.98 ± 0.19 | 2.02 | 9.6 | 17.3 | 2.6 | -3 | 781 |
| 3 | Hru&Jav | -22.43 ± 0.33 | 3.21 | 2.76 ± 0.42 | 4.30 | 8.4 | 17.7 | -1.1 | 562 | 1806 |
| 4 | G. Pomerania | -22.42 ± 0.09 | 1.53 | 2.78 ± 0.36 | 7.21 | 7.3 | 16.4 | -1.7 | 99 | 657 |
| 5 | W. Pomerania | -22.66 ± 0.24 | 2.21 | 5.06 ± 0.23 | 1.91 | 8.5 | 17.8 | -0.7 | 16 | 602 |
| 6 | Goleniów | -22.84 ± 0.18 | 1.78 | 4.25 ± 0.37 | 5.45 | 8.6 | 18.0 | -0.9 | 38 | 567 |
| 7 | Bardo | -22.33 ± 0.27 | 3.54 | 3.56 ± 0.27 | 3.03 | 7.3 | 16.4 | -1.5 | 490 | 614 |
| 8 | Piorków | -22.57 ± 0.10 | 1.36 | 3.16 ± 0.30 | 3.79 | 8.0 | 17.9 | -2.7 | 199 | 549 |
| 9 | Dukla | -22.95 ± 0.09 | 1.34 | 2.75 ± 0.10 | 1.77 | 6.9 | 16.3 | -3.2 | 492 | 789 |
| 10 | Ustrzyki | -23.13 ± 0.08 | 1.85 | 2.90 ± 0.08 | 1.42 | 6.6 | 15.8 | -3.1 | 530 | 835 |
| 11 | Chełm | -21.81 ± 0.21 | 3.12 | 4.63 ± 0.19 | 2.42 | 7.5 | 17.7 | -3.2 | 173 | 552 |
| 12 | Włodawa | -22.88 ± 0.09 | 0.82 | 3.40 ± 0.29 | 3.13 | 7.5 | 17.7 | -3.2 | 181 | 529 |
| 13 | Białowieża | -22.92 ± 0.14 | 3.33 | 3.31 ± 0.35 | 5.59 | 6.8 | 17.3 | -3.7 | 172 | 593 |
| 14 | Knyszyn | -22.48 ± 0.16 | 3.15 | 3.71 ± 0.42 | 7.00 | 6.6 | 17.2 | -4.1 | 167 | 589 |
| 15 | Augustów | -22.86 ± 0.21 | 3.07 | 2.93 ± 0.32 | 4.14 | 6.7 | 17.3 | -3.5 | 136 | 582 |
