# Supplementary material for: Environmental factors shaping stable isotope signatures of modern red deer (Cervus elaphus) inhabiting various habitats
Source: PLoS One. 2021 Aug 13;16(8):e0255398. doi: 10.1371/journal.pone.0255398 (PMC8362983; doi:10.1371/journal.pone.0255398)
Supplement: S3 Table — (DOCX) [file pone.0255398.s003.docx]

**Environmental factors shaping stable isotope signatures of modern red deer (*Cervus elaphus)* inhabiting various habitats**

Maciej Sykut*, Sławomira Pawełczyk, Tomasz Borowik, Boštjan Pokorny, Katarina Flajšman, Tjibbe Hunink, Magdalena Niedziałkowska

Corresponding author: Maciej Sykut mail: msykut@ibs.bialowieza.pl

S3 Table. Values of *δ*^13^C and *δ*^15^N measured in red deer bones collagen of red deer bones, carbon to nitrogen atomic ratio (C/N), percentage of carbon (%C) and nitrogen (%N) in a sample, year of death of sampled red deer individual (year), coordinates (y, x), the mean annual temperature (annual temp.), the mean July temperature (July temp.), the mean January temperature (Jan temp.), the altitude, the annual precipitation (prec), distance to the water area (sea dist.) *samples with *δ*^13^C and *δ*^15^N values recalculated by the formulas described in the Sykut et al.[1]; *δ*^13^Ccorr: *δ*^13^C values corrected for the shift in *δ*^13^C values (Atm_corr) caused by anthropogenic CO_2_ emissions by the formula proposed by Feng [2].

| Individual ID | Site | *δ* ^13^C | Atm corr | *δ* ^13^Ccorr | *δ* ^15^N | C/N | %C | %N | year | y | x | annual temp. (°C) | July temp. (°C) | Jan temp. (°C) | altitude m a.s.l. | prec. (mm) | sea dist. (km) |
| --- | --- | --- | --- | --- | --- | --- | --- | --- | --- | --- | --- | --- | --- | --- | --- | --- | --- |
| 320B | Augustów | -24.55 | 2.10 | -22.45 | 1.66 | 3.3 | 46.2 | 16.3 | 2010 | 53.927 | 23.254 | 6.7 | 17.3 | -3.6 | 134 | 585 | - |
| 321B | Augustów | -24.82 | 2.10 | -22.71 | 4.78 | 3.3 | 47.5 | 16.7 | 2010 | 53.920 | 23.382 | 6.7 | 17.3 | -3.5 | 116 | 584 | - |
| 322B | Augustów | -24.68 | 2.10 | -22.58 | 3.63 | 3.4 | 47.1 | 16.1 | 2010 | 53.866 | 23.245 | 6.7 | 17.3 | -3.5 | 143 | 585 | - |
| 323BC | Augustów | -24.49 | 2.15 | -22.34 | 4.27 | 3.4 | 44.9 | 15.6 | 2011 | 53.894 | 23.141 | 6.8 | 17.3 | -3.6 | 143 | 578 | - |
| 324BC | Augustów | -24.29 | 2.10 | -22.19 | 3.26 | 3.4 | 42.9 | 14.7 | 2010 | 53.880 | 23.150 | 6.8 | 17.3 | -3.6 | 141 | 578 | - |
| 325BC | Augustów | -24.37 | 2.10 | -22.27 | 3.06 | 3.4 | 45.7 | 15.8 | 2010 | 53.983 | 23.021 | 6.8 | 17.3 | -3.5 | 139 | 578 | - |
| 326B | Augustów | -24.74 | 2.10 | -22.64 | 4.34 | 3.4 | 42.6 | 14.5 | 2010 | 53.880 | 23.250 | 6.7 | 17.3 | -3.6 | 147 | 585 | - |
| 328 BC | Augustów | -25.01 | 2.10 | -22.90 | 4.92 | 3.2 | 44.1 | 15.9 | 2010 | 53.900 | 22.977 | 6.7 | 17.4 | -3.5 | 134 | 577 | - |
| 330BC | Augustów | -24.33 | 2.08 | -22.25 | 2.63 | 3.2 | 43.2 | 15.6 | 2009/10 | 53.920 | 23.068 | 6.8 | 17.3 | -3.5 | 140 | 578 | - |
| 335B | Augustów | -26.10 | 2.06 | -24.05 | 0.78 | 3.4 | 46.4 | 15.7 | 2009 | 53.954 | 23.282 | 6.7 | 17.3 | -3.6 | 129 | 585 | - |
| 338 BA | Augustów | -24.43 | 2.01 | -22.42 | 2.14 | 3.2 | 45.8 | 16.7 | 2008 | 53.944 | 23.097 | 6.8 | 17.3 | -3.5 | 137 | 578 | - |
| 339 B | Augustów | -23.82 | 2.06 | -21.76 | 2.70 | 3.4 | 46.8 | 16.0 | 2009 | 53.984 | 23.239 | 6.7 | 17.3 | -3.5 | 133 | 585 | - |
| 341BS | Augustów | -26.30 | 2.06 | -24.25 | 0.96 | 3.3 | 45.6 | 16.1 | 2009 | 53.987 | 23.213 | 6.7 | 17.3 | -3.5 | 139 | 585 | - |
| 343BS | Augustów | -25.44 | 2.13 | -23.31 | 1.63 | 3.5 | 47.0 | 15.8 | 2010/11 | 53.873 | 23.260 | 6.7 | 17.3 | -3.6 | 142 | 585 | - |
| 345BS | Augustów | -26.93 | 2.10 | -24.83 | 3.14 | 3.4 | 45.6 | 15.4 | 2010 | 53.902 | 23.364 | 6.7 | 17.3 | -3.6 | 123 | 584 | - |
| 351BC | Augustów | -24.93 | 2.10 | -22.83 | 2.98 | 3.4 | 44.4 | 15.2 | 2010 | 53.884 | 23.291 | 6.7 | 17.3 | -3.6 | 140 | 585 | - |
| 45WN* | Bardo | -25.05 | 2.34 | -22.71 | 3.57 | 3.7 | 43.6 | 13.7 | 2015 | 50.465 | 16.923 | 7.3 | 17.2 | -1.0 | 267 | 618 | - |
| 46WN* | Bardo | -25.13 | 2.34 | -22.79 | 2.64 | 3.6 | 42.9 | 13.9 | 2015 | 50.427 | 16.795 | 7.2 | 16.2 | -1.8 | 484 | 604 | - |
| 47WN* | Bardo | -25.73 | 2.34 | -23.38 | 2.43 | 3.6 | 41.9 | 13.5 | 2015 | 50.427 | 16.795 | 7.2 | 16.2 | -1.8 | 484 | 604 | - |
| 49WN* | Bardo | -24.80 | 2.34 | -22.45 | 4.88 | 3.7 | 43.4 | 13.7 | 2015 | 50.418 | 16.790 | 7.2 | 16.0 | -1.9 | 553 | 604 | - |
| 51WN* | Bardo | -25.02 | 2.34 | -22.68 | 3.73 | 3.7 | 43.2 | 13.6 | 2015 | 50.421 | 16.839 | 7.3 | 16.2 | -1.6 | 630 | 618 | - |
| 52WN* | Bardo | -25.30 | 2.34 | -22.96 | 2.73 | 3.7 | 43.5 | 13.7 | 2015 | 50.421 | 16.839 | 7.3 | 16.2 | -1.6 | 630 | 618 | - |
| 53WN* | Bardo | -25.60 | 2.34 | -23.26 | 2.74 | 3.9 | 43.6 | 13.2 | 2015 | 50.427 | 16.795 | 7.2 | 16.2 | -1.8 | 484 | 604 | - |
| 56WN* | Bardo | -25.17 | 2.34 | -22.83 | 2.78 | 4.0 | 43.7 | 12.9 | 2015 | 50.473 | 16.823 | 7.2 | 16.6 | -1.3 | 382 | 604 | - |
| 57WN* | Bardo | -24.72 | 2.34 | -22.38 | 3.88 | 3.8 | 42.5 | 13.0 | 2015 | 50.421 | 16.839 | 7.3 | 16.2 | -1.6 | 630 | 618 | - |
| 58WN* | Bardo | -25.05 | 2.34 | -22.71 | 2.64 | 3.9 | 35.9 | 10.6 | 2015 | 50.421 | 16.839 | 7.3 | 16.2 | -1.6 | 630 | 618 | - |
| 59WN* | Bardo | -24.46 | 2.34 | -22.12 | 2.70 | 3.9 | 41.4 | 12.5 | 2015 | 50.465 | 16.923 | 7.3 | 17.2 | -1.0 | 267 | 618 | - |
| 60WN* | Bardo | -22.71 | 2.34 | -20.36 | 5.26 | 4.0 | 41.0 | 12.1 | 2015 | 50.471 | 16.941 | 7.3 | 17.2 | -1.0 | 253 | 618 | - |
| 61WN* | Bardo | -24.97 | 2.34 | -22.63 | 3.92 | 4.2 | 41.5 | 11.6 | 2015 | 50.421 | 16.839 | 7.3 | 16.2 | -1.6 | 630 | 618 | - |
| 62WN* | Bardo | -22.94 | 2.34 | -20.60 | 5.04 | 3.8 | 42.2 | 12.8 | 2015 | 50.497 | 16.857 | 7.3 | 16.9 | -1.2 | 261 | 618 | - |
| 63WN* | Bardo | -25.82 | 2.34 | -23.48 | 2.63 | 4.2 | 43.7 | 12.0 | 2015 | 50.421 | 16.839 | 7.3 | 16.2 | -1.6 | 630 | 618 | - |
| 64WN* | Bardo | -22.28 | 2.34 | -19.94 | 5.46 | 3.9 | 42.9 | 12.9 | 2015 | 50.421 | 16.839 | 7.3 | 16.2 | -1.6 | 630 | 618 | - |
| 198B | Białowieża | -22.88 | 0.83 | -22.05 | 4.25 | 3.3 | 44.8 | 16.1 | 1967 | 52.733 | 23.868 | 6.7 | 17.3 | -3.6 | 185 | 596 | - |
| 200B | Białowieża | -23.36 | 0.85 | -22.52 | 4.54 | 3.3 | 45.9 | 16.3 | 1968 | 52.822 | 23.827 | 6.8 | 17.3 | -4.0 | 148 | 594 | - |
| 236B | Białowieża | -24.72 | 2.39 | -22.33 | 6.2 | 3.3 | 45.6 | 16.3 | 2016 | 52.612 | 23.454 | 6.9 | 17.3 | -3.9 | 180 | 590 | - |
| 242B | Białowieża | -25.14 | 2.45 | -22.69 | 5.69 | 3.3 | 45.9 | 16.4 | 2017 | 52.653 | 23.551 | 6.8 | 17.3 | -3.7 | 177 | 592 | - |
| 243B | Białowieża | -25.40 | 2.45 | -22.95 | 6.02 | 3.5 | 45.0 | 14.9 | 2017 | 52.656 | 23.557 | 6.8 | 17.3 | -3.7 | 177 | 592 | - |
| 244B | Białowieża | -25.11 | 2.45 | -22.66 | 3.72 | 3.3 | 45.3 | 16.2 | 2017 | 52.851 | 23.893 | 6.6 | 17.2 | -4.0 | 163 | 591 | - |
| 245B | Białowieża | -25.84 | 2.45 | -23.39 | 1.00 | 3.3 | 46.9 | 16.6 | 2017 | 52.811 | 23.774 | 6.8 | 17.3 | -4.0 | 169 | 594 | - |
| 246B | Białowieża | -25.55 | 2.45 | -23.11 | 0.67 | 3.6 | 46.7 | 15.3 | 2017 | 52.809 | 23.698 | 6.8 | 17.3 | -3.8 | 181 | 594 | - |
| 247B | Białowieża | -24.67 | 2.45 | -22.22 | 5.93 | 3.4 | 46.4 | 16.1 | 2017 | 52.809 | 23.698 | 6.8 | 17.3 | -3.8 | 181 | 594 | - |
| 248B | Białowieża | -23.11 | 2.45 | -20.66 | 4.69 | 3.0 | 44.3 | 17.5 | 2017 | 52.809 | 23.698 | 6.8 | 17.3 | -3.8 | 181 | 594 | - |
| 305B | Białowieża | -24.70 | 2.01 | -22.68 | 5.44 | 2.9 | 44.9 | 17.7 | 2008 | 52.697 | 23.595 | 6.8 | 17.3 | -3.6 | 168 | 594 | - |
| 318B | Białowieża | -24.61 | 2.06 | -22.55 | 1.00 | 3.6 | 46.1 | 15.0 | 2009 | 52.651 | 23.630 | 6.8 | 17.4 | -3.5 | 163 | 592 | - |
| 319B | Białowieża | -24.61 | 2.15 | -22.46 | 2.16 | 3.2 | 45.5 | 16.4 | 2011 | 52.735 | 23.662 | 6.8 | 17.2 | -3.6 | 176 | 594 | - |
| 329B | Białowieża | -25.57 | 2.10 | -23.46 | 2.36 | 3.1 | 43.6 | 16.5 | 2010 | 52.711 | 23.775 | 6.8 | 17.3 | -3.6 | 172 | 594 | - |
| 331B | Białowieża | -25.37 | 2.06 | -23.31 | 2.06 | 3.1 | 44.5 | 17.0 | 2009 | 52.642 | 23.621 | 6.8 | 17.3 | -3.5 | 163 | 592 | - |
| 332B | Białowieża | -26.05 | 2.06 | -24.00 | 4.44 | 3.0 | 43.3 | 16.6 | 2009 | 52.691 | 23.688 | 6.8 | 17.3 | -3.4 | 177 | 594 | - |
| 333B | Białowieża | -25.89 | 2.06 | -23.83 | 1.97 | 3.4 | 43.7 | 15.2 | 2009 | 52.654 | 23.713 | 6.8 | 17.4 | -3.4 | 166 | 596 | - |
| 334B | Białowieża | -25.34 | 2.06 | -23.28 | 2.91 | 3.3 | 43.2 | 15.4 | 2009 | 52.649 | 23.704 | 6.8 | 17.4 | -3.4 | 162 | 596 | - |
| 336B | Białowieża | -25.44 | 2.06 | -23.38 | 3.05 | 3.3 | 43.9 | 15.6 | 2009 | 52.767 | 23.713 | 6.8 | 17.3 | -3.7 | 176 | 594 | - |
| 337BC | Białowieża | -25.01 | 2.06 | -22.95 | 3.43 | 3.0 | 42.6 | 16.6 | 2009 | 52.661 | 23.631 | 6.8 | 17.4 | -3.5 | 163 | 592 | - |
| 340B | Białowieża | -24.79 | 1.97 | -22.82 | 4.57 | 3.3 | 45.4 | 16.3 | 2007 | 52.906 | 23.643 | 6.8 | 17.3 | -3.8 | 149 | 587 | - |
| 342BS | Białowieża | -25.22 | 2.01 | -22.77 | 3.81 | 3.3 | 45.3 | 16.1 | 2008 | 52.697 | 23.733 | 6.8 | 17.3 | -3.6 | 182 | 594 | - |
| 346BS | Białowieża | -26.05 | 2.10 | -23.95 | 0.61 | 3.5 | 43.7 | 14.5 | 2010 | 52.706 | 23.665 | 6.8 | 17.3 | -3.5 | 175 | 594 | - |
| 347BS | Białowieża | -25.49 | 2.06 | -23.43 | 1.89 | 3.3 | 44.0 | 15.8 | 2009 | 52.764 | 23.665 | 6.8 | 17.2 | -3.8 | 176 | 594 | - |
| 348BS | Białowieża | -24.94 | 2.06 | -22.88 | 0.96 | 3.4 | 44.4 | 15.4 | 2009 | 52.698 | 23.728 | 6.8 | 17.3 | -3.6 | 182 | 594 | - |
| 349BS | Białowieża | -25.56 | 2.01 | -23.54 | 2.71 | 3.4 | 44.4 | 15.4 | 2008 | 52.695 | 23.774 | 6.8 | 17.3 | -3.6 | 187 | 594 | - |
| 11C* | Chełm | -23.95 | 2.34 | -21.61 | 4.06 | 3.8 | 29.1 | 8.8 | 2015 | 51.153 | 23.676 | 7.5 | 17.6 | -3.2 | 177 | 570 | - |
| 16C* | Chełm | -24.13 | 2.34 | -21.79 | 4.99 | 4.0 | 30.8 | 9.0 | 2015 | 51.187 | 23.606 | 7.5 | 17.7 | -3.1 | 175 | 547 | - |
| 18C* | Chełm | -24.74 | 2.34 | -22.39 | 3.79 | 4.0 | 31.7 | 9.2 | 2015 | 51.253 | 23.654 | 7.5 | 17.8 | -3.4 | 165 | 547 | - |
| 19CP* | Chełm | -24.81 | 2.34 | -22.47 | 3.93 | 3.6 | 42.9 | 14.1 | 2015 | 51.225 | 23.517 | 7.5 | 17.6 | -3.1 | 175 | 547 | - |
| 213CP* | Chełm | -24.82 | 2.39 | -22.42 | 3.97 | 3.5 | 47.9 | 15.8 | 2016 | 51.302 | 23.605 | 7.5 | 17.7 | -3.4 | 169 | 547 | - |
| 216CP* | Chełm | -24.88 | 2.39 | -22.49 | 4.69 | 3.5 | 46.1 | 15.3 | 2016 | 51.226 | 23.479 | 7.5 | 17.8 | -3.1 | 173 | 543 | - |
| 217CP* | Chełm | -24.32 | 2.39 | -21.92 | 4.04 | 3.6 | 41.8 | 13.6 | 2016 | 51.255 | 23.453 | 7.5 | 17.8 | -3.3 | 171 | 543 | - |
| 218CP* | Chełm | -21.90 | 2.39 | -19.50 | 6.21 | 3.5 | 42.5 | 14.1 | 2016 | 51.157 | 23.694 | 7.5 | 17.6 | -3.2 | 172 | 570 | - |
| 219 CP* | Chełm | -24.72 | 2.39 | -22.33 | 4.84 | 3.5 | 43.2 | 14.6 | 2016 | 51.211 | 23.692 | 7.5 | 17.7 | -3.3 | 172 | 558 | - |
| 21CZ* | Chełm | -24.23 | 2.34 | -21.89 | 5.55 | 3.3 | 42.2 | 14.9 | 2015 | 51.225 | 23.494 | 7.5 | 17.8 | -3.1 | 170 | 543 | - |
| 221CP* | Chełm | -24.71 | 2.39 | -22.31 | 5.75 | 3.8 | 45.2 | 13.9 | 2016 | 51.198 | 23.636 | 7.5 | 17.6 | -3.1 | 177 | 547 | - |
| 2C* | Chełm | -23.24 | 2.34 | -20.89 | 5.35 | 4.0 | 34.2 | 10.1 | 2015 | 51.187 | 23.606 | 7.5 | 17.7 | -3.1 | 175 | 547 | - |
| 6C* | Chełm | -23.22 | 2.34 | -20.88 | 4.22 | 3.6 | 29.7 | 9.6 | 2015 | 51.153 | 23.676 | 7.5 | 17.6 | -3.2 | 177 | 570 | - |
| 7C* | Chełm | -23.65 | 2.34 | -21.31 | 4.08 | 4.0 | 30.8 | 9.0 | 2015 | 51.067 | 23.748 | 7.5 | 17.6 | -3.2 | 183 | 570 | - |
| 8CP* | Chełm | -24.52 | 2.34 | -22.17 | 4.08 | 3.5 | 45.6 | 15.0 | 2015 | 51.225 | 23.494 | 7.5 | 17.8 | -3.1 | 170 | 543 | - |
| 9C* | Chełm | -24.97 | 2.34 | -22.63 | 4.56 | 4.1 | 30.2 | 8.5 | 2015 | 51.255 | 23.453 | 7.5 | 17.8 | -3.3 | 171 | 543 | - |
| 281D* | Dukla | -24.86 | 2.45 | -22.41 | 2.42 | 3.6 | 31.9 | 10.4 | 2017 | 49.482 | 21.695 | 6.7 | 16.5 | -3.2 | 428 | 808 | - |
| 282D* | Dukla | -25.88 | 2.45 | -23.43 | 1.68 | 3.6 | 34.1 | 11.0 | 2017 | 49.442 | 21.766 | 6.7 | 15.9 | -3.3 | 585 | 808 | - |
| 283D* | Dukla | -25.32 | 2.45 | -22.87 | 3.26 | 3.6 | 33.7 | 10.8 | 2017 | 49.435 | 21.734 | 6.7 | 16.1 | -3.3 | 591 | 808 | - |
| 284D* | Dukla | -24.99 | 2.45 | -22.55 | 3.42 | 3.5 | 32.4 | 10.8 | 2017 | 49.445 | 21.707 | 6.7 | 16.4 | -3.3 | 449 | 808 | - |
| 285D* | Dukla | -24.95 | 2.45 | -22.50 | 3.12 | 3.7 | 30.9 | 9.8 | 2017 | 49.533 | 21.676 | 7.5 | 16.6 | -3.0 | 356 | 742 | - |
| 286D* | Dukla | -25.43 | 2.45 | -22.98 | 3.03 | 3.6 | 31.7 | 10.3 | 2017 | 49.482 | 21.695 | 6.7 | 16.5 | -3.2 | 428 | 808 | - |
| 287D* | Dukla | -25.89 | 2.45 | -23.45 | 2.41 | 3.6 | 31.8 | 10.3 | 2017 | 49.502 | 21.671 | 7.5 | 16.6 | -3.0 | 523 | 742 | - |
| 67D* | Dukla | -25.06 | 2.34 | -22.72 | 3.15 | 3.5 | 32.6 | 10.8 | 2015 | 49.533 | 21.676 | 7.5 | 16.6 | -3.0 | 356 | 742 | - |
| 68D* | Dukla | -25.08 | 2.34 | -22.73 | 3.45 | 3.7 | 32.2 | 10.2 | 2015 | 49.502 | 21.671 | 7.5 | 16.6 | -3.0 | 523 | 742 | - |
| 70D* | Dukla | -25.30 | 2.34 | -22.95 | 3.02 | 3.6 | 28.6 | 9.3 | 2015 | 49.502 | 21.671 | 7.5 | 16.6 | -3.0 | 523 | 742 | - |
| 71D* | Dukla | -25.00 | 2.45 | -22.55 | 2.54 | 3.5 | 33.3 | 11.2 | 2017 | 49.458 | 21.721 | 6.7 | 16.1 | -3.3 | 482 | 808 | - |
| 72D* | Dukla | -25.07 | 2.34 | -22.73 | 2.45 | 3.6 | 33.5 | 11.0 | 2015 | 49.502 | 21.671 | 7.5 | 16.6 | -3.0 | 523 | 742 | - |
| 73D* | Dukla | -25.31 | 2.45 | -22.86 | 3.06 | 4.0 | 27.9 | 8.2 | 2017 | 49.435 | 21.734 | 6.7 | 16.1 | -3.3 | 591 | 808 | - |
| 74D* | Dukla | -25.85 | 2.34 | -23.51 | 3.06 | 3.7 | 31.1 | 9.8 | 2015 | 49.498 | 21.685 | 6.7 | 16.5 | -3.2 | 414 | 808 | - |
| 75D* | Dukla | -24.56 | 2.34 | -22.21 | 2.84 | 3.5 | 30.7 | 10.3 | 2015 | 49.498 | 21.685 | 6.7 | 16.5 | -3.2 | 414 | 808 | - |
| 76D* | Dukla | -24.85 | 2.34 | -22.50 | 2.50 | 3.6 | 32.4 | 10.4 | 2015 | 49.442 | 21.766 | 6.7 | 15.9 | -3.3 | 585 | 808 | - |
| 77D* | Dukla | -26.00 | 2.45 | -23.55 | 2.22 | 3.7 | 30.7 | 9.8 | 2017 | 49.458 | 21.721 | 6.7 | 16.1 | -3.3 | 482 | 808 | - |
| 78D* | Dukla | -25.60 | 2.34 | -23.26 | 2.76 | 3.7 | 33.2 | 10.5 | 2015 | 49.435 | 21.734 | 6.7 | 16.1 | -3.3 | 591 | 808 | - |
| 79DD* | Dukla | -26.00 | 2.45 | -23.55 | 2.35 | 4.3 | 28.9 | 7.9 | 2017 | 49.473 | 21.624 | 6.9 | 16.2 | -3.3 | 542 | 785 | - |
| 80D* | Dukla | -25.75 | 2.34 | -23.41 | 2.13 | 3.7 | 32.3 | 10.1 | 2015 | 49.442 | 21.766 | 6.7 | 15.9 | -3.3 | 585 | 808 | - |
| 81D* | Dukla | -25.41 | 2.34 | -23.07 | 3.05 | 3.6 | 34.8 | 11.2 | 2015 | 49.498 | 21.685 | 6.7 | 16.5 | -3.2 | 414 | 808 | - |
| 83D* | Dukla | -25.41 | 2.34 | -23.07 | 2.60 | 3.8 | 33.5 | 10.2 | 2015 | 49.482 | 21.695 | 6.7 | 16.5 | -3.2 | 428 | 808 | - |
| 422N | Flevoland | -24.78 | 2.56 | -22.22 | 8.32 | 3.2 | 44.5 | 16.3 | 2019 | 52.444 | 5.365 | 9.6 | 17.3 | 2.6 | -3 | 781 | 2 |
| 423N | Flevoland | -25.11 | 2.56 | -22.55 | 8.47 | 3.2 | 44.2 | 15.9 | 2019 | 52.444 | 5.365 | 9.6 | 17.3 | 2.6 | -3 | 781 | 2 |
| 424N | Flevoland | -24.29 | 2.56 | -21.73 | 9.29 | 3.2 | 41.8 | 15.3 | 2019 | 52.444 | 5.365 | 9.6 | 17.3 | 2.6 | -3 | 781 | 2 |
| 425N | Flevoland | -24.49 | 2.56 | -21.94 | 8.20 | 3.4 | 44.4 | 15.4 | 2019 | 52.444 | 5.365 | 9.6 | 17.3 | 2.6 | -3 | 781 | 2 |
| 426N | Flevoland | -24.63 | 2.56 | -22.07 | 7.47 | 3.3 | 43.1 | 15.4 | 2019 | 52.444 | 5.365 | 9.6 | 17.3 | 2.6 | -3 | 781 | 2 |
| 427N | Flevoland | -24.75 | 2.56 | -22.19 | 7.84 | 3.4 | 44.7 | 15.5 | 2019 | 52.444 | 5.365 | 9.6 | 17.3 | 2.6 | -3 | 781 | 2 |
| 428N | Flevoland | -24.32 | 2.56 | -21.76 | 8.51 | 3.3 | 40.0 | 14.2 | 2019 | 52.444 | 5.365 | 9.6 | 17.3 | 2.6 | -3 | 781 | 2 |
| 429N | Flevoland | -24.39 | 2.56 | -21.84 | 7.69 | 3.4 | 42.9 | 14.9 | 2019 | 52.444 | 5.365 | 9.6 | 17.3 | 2.6 | -3 | 781 | 2 |
| 430N | Flevoland | -24.45 | 2.56 | -21.89 | 7.27 | 3.2 | 42.3 | 15.2 | 2019 | 52.444 | 5.365 | 9.6 | 17.3 | 2.6 | -3 | 781 | 2 |
| 431N | Flevoland | -24.82 | 2.56 | -22.26 | 7.36 | 3.2 | 43.4 | 15.7 | 2019 | 52.444 | 5.365 | 9.6 | 17.3 | 2.6 | -3 | 781 | 2 |
| 432N | Flevoland | -24.39 | 2.56 | -21.84 | 7.33 | 3.3 | 41.7 | 14.9 | 2019 | 52.444 | 5.365 | 9.6 | 17.3 | 2.6 | -3 | 781 | 2 |
| 24G | G. Pomerania | -24.85 | 2.34 | -22.50 | 1.60 | 3.4 | 45.0 | 15.5 | 2015 | 54.665 | 18.197 | 7.5 | 16.6 | -0.8 | 80 | 663 | 19 |
| 25G | G. Pomerania | -25.29 | 2.34 | -22.95 | 1.54 | 3.4 | 45.9 | 15.8 | 2015 | 54.591 | 18.034 | 7.4 | 16.4 | -1.2 | 56 | 656 | 27 |
| 26G | G. Pomerania | -24.52 | 2.34 | -22.18 | 1.43 | 3.4 | 44.4 | 15.2 | 2015 | 54.620 | 17.901 | 7.3 | 16.5 | -1.7 | 111 | 665 | 23 |
| 28G | G. Pomerania | -25.19 | 2.34 | -22.84 | 0.62 | 3.4 | 46.2 | 16.0 | 2015 | 54.490 | 17.970 | 6.8 | 16.3 | -2.4 | 131 | 678 | 38 |
| 29G | G. Pomerania | -24.29 | 2.34 | -21.95 | 0.86 | 3.5 | 46.1 | 15.5 | 2015 | 54.599 | 17.927 | 7.3 | 16.4 | -1.4 | 92 | 665 | 25 |
| 30G | G. Pomerania | -25.34 | 2.34 | -23.00 | 3.12 | 3.3 | 46.6 | 16.5 | 2015 | 54.599 | 17.927 | 7.3 | 16.4 | -1.4 | 92 | 665 | 25 |
| 31G | G. Pomerania | -25.32 | 2.34 | -22.97 | 2.84 | 3.3 | 46.5 | 16.6 | 2015 | 54.635 | 17.904 | 7.3 | 16.3 | -1.5 | 116 | 665 | 21 |
| 32G | G. Pomerania | -24.39 | 2.34 | -22.05 | 3.23 | 3.3 | 48.2 | 17.0 | 2015 | 54.674 | 17.971 | 7.5 | 16.4 | -1.1 | 119 | 638 | 18 |
| 33G | G. Pomerania | -24.56 | 2.34 | -22.22 | 4.39 | 3.3 | 45.2 | 15.9 | 2015 | 54.592 | 17.847 | 7.3 | 16.6 | -1.7 | 63 | 665 | 25 |
| 34G | G. Pomerania | -24.42 | 2.34 | -22.08 | 4.41 | 3.3 | 45.8 | 16.2 | 2015 | 54.566 | 17.957 | 7.3 | 16.4 | -1.6 | 67 | 665 | 29 |
| 35G | G. Pomerania | -25.39 | 2.34 | -23.05 | 3.53 | 3.3 | 45.9 | 16.1 | 2015 | 54.440 | 18.090 | 6.8 | 16.2 | -2.6 | 177 | 677 | 31 |
| 38G | G. Pomerania | -24.93 | 2.34 | -22.58 | 2.22 | 3.4 | 46.9 | 16.1 | 2015 | 54.533 | 17.942 | 7.3 | 16.2 | -2.2 | 163 | 665 | 33 |
| 39G | G. Pomerania | -24.45 | 2.34 | -22.10 | 1.72 | 3.4 | 46.4 | 15.8 | 2015 | 54.592 | 17.962 | 7.3 | 16.3 | -1.2 | 106 | 665 | 27 |
| 40G | G. Pomerania | -24.51 | 2.34 | -22.16 | 1.60 | 3.4 | 46.7 | 16.2 | 2015 | 54.659 | 17.878 | 7.3 | 16.3 | -1.5 | 123 | 665 | 18 |
| 417SP | G. Pomerania | -24.36 | 2.50 | -21.86 | 3.35 | 3.5 | 44.7 | 14.8 | 2018 | 54.342 | 17.475 | 7.3 | 16.3 | -2.4 | 107 | 633 | 38 |
| 418SP | G. Pomerania | -24.95 | 2.50 | -22.45 | 2.55 | 3.5 | 47.4 | 15.9 | 2018 | 54.364 | 17.302 | 7.4 | 16.5 | -2.3 | 79 | 628 | 31 |
| 419SP | G. Pomerania | -25.18 | 2.50 | -22.68 | 5.63 | 3.3 | 46.1 | 16.1 | 2018 | 54.651 | 17.222 | 7.6 | 16.7 | -0.4 | 5 | 617 | 7 |
| 41G | G. Pomerania | -25.44 | 2.34 | -23.09 | 1.17 | 3.4 | 45.9 | 15.8 | 2015 | 54.541 | 18.000 | 7.3 | 16.2 | -2.1 | 102 | 665 | 32 |
| 420SP | G. Pomerania | -24.50 | 2.50 | -22.00 | 3.76 | 3.5 | 46.2 | 15.6 | 2018 | 54.326 | 17.471 | 7.1 | 16.4 | -2.5 | 115 | 628 | 39 |
| 421SP | G. Pomerania | -24.07 | 2.50 | -21.56 | 7.83 | 3.2 | 46.5 | 17.1 | 2018 | 54.651 | 17.222 | 7.6 | 16.7 | -0.4 | 5 | 617 | 7 |
| 42G | G. Pomerania | -24.65 | 2.34 | -22.31 | 2.64 | 3.5 | 45.8 | 15.3 | 2015 | 54.490 | 18.103 | 6.8 | 16.3 | -2.3 | 136 | 677 | 30 |
| 43G | G. Pomerania | -24.90 | 2.34 | -22.56 | 0.66 | 3.4 | 45.3 | 15.4 | 2015 | 54.490 | 18.103 | 6.8 | 16.3 | -2.3 | 136 | 677 | 28 |
| 44G | G. Pomerania | -24.93 | 2.34 | -22.58 | 3.25 | 3.5 | 44.9 | 15.2 | 2015 | 54.570 | 17.877 | 7.3 | 16.5 | -1.7 | 103 | 665 | 28 |
| L1971* | Hru&Jav | -25.32 | 2.06 | -23.27 | 0.65 | 3.5 | 45.8 | 15.5 | 2009 | 45.923 | 13.975 | 8.9 | 16.9 | -1.8 | 994 | 2041 | - |
| L1977* | Hru&Jav | -24.50 | 2.06 | -22.44 | 2.52 | 3.2 | 47.5 | 17.0 | 2009 | 45.861 | 14.118 | 7.7 | 16.6 | -1.6 | 772 | 2097 | - |
| L24132* | Hru&Jav | -22.85 | 2.06 | -20.80 | 2.57 | 3.2 | 47.3 | 17.4 | 2009 | 45.726 | 14.171 | 8.3 | 18.0 | -0.3 | 516 | 1653 | - |
| L24136* | Hru&Jav | -24.47 | 2.06 | -22.41 | 4.90 | 3.4 | 44.4 | 15.3 | 2009 | 45.735 | 14.120 | 8.7 | 17.5 | 0.1 | 618 | 1773 | - |
| L2579* | Hru&Jav | -23.00 | 2.06 | -20.94 | 3.86 | 3.3 | 46.3 | 16.2 | 2009 | 45.798 | 14.170 | 8.3 | 17.6 | -0.8 | 542 | 1653 | - |
| L2584* | Hru&Jav | -23.22 | 2.06 | -21.16 | 4.85 | 3.3 | 47.8 | 17.1 | 2009 | 45.798 | 14.170 | 8.3 | 17.6 | -0.8 | 542 | 1653 | - |
| L2756* | Hru&Jav | -26.03 | 2.06 | -23.97 | 1.78 | 3.6 | 48.4 | 15.8 | 2009 | 45.862 | 14.247 | 8.6 | 17.3 | -1.1 | 453 | 1741 | - |
| L2788* | Hru&Jav | -25.29 | 2.06 | -23.23 | 2.65 | 3.4 | 44.8 | 15.4 | 2009 | 45.871 | 14.246 | 8.6 | 17.3 | -1.1 | 448 | 1741 | - |
| L296* | Hru&Jav | -25.13 | 2.06 | -23.07 | 0.60 | 3.3 | 47.1 | 16.9 | 2009 | 45.879 | 14.169 | 8.6 | 17.8 | -1.6 | 666 | 1741 | - |
| L2983* | Hru&Jav | -23.45 | 2.06 | -21.39 | 1.61 | 3.3 | 47.7 | 16.9 | 2009 | 45.906 | 14.156 | 7.7 | 17.4 | -1.7 | 502 | 2097 | - |
| L3116* | Hru&Jav | -26.07 | 2.06 | -24.01 | 3.54 | 3.3 | 45.8 | 16.0 | 2009 | 45.952 | 14.271 | 8.6 | 18.4 | -1.2 | 405 | 1741 | - |
| L3173* | Hru&Jav | -24.57 | 2.06 | -22.51 | 3.56 | 3.5 | 48.3 | 16.1 | 2009 | 45.979 | 14.322 | 8.6 | 19.9 | -1.1 | 283 | 1741 | - |
| 225B | Knyszyn | -24.98 | 2.39 | -22.59 | 3.31 | 3.3 | 46.4 | 16.2 | 2016 | 53.224 | 23.620 | 6.6 | 17.3 | -3.8 | 165 | 588 | - |
| 226B | Knyszyn | -25.45 | 2.39 | -23.05 | 1.97 | 3.3 | 46.3 | 16.3 | 2016 | 53.224 | 23.620 | 6.6 | 17.3 | -3.8 | 165 | 588 | - |
| 227B | Knyszyn | -24.95 | 2.39 | -22.55 | 4.08 | 3.3 | 46.7 | 16.4 | 2016 | 53.181 | 23.792 | 6.5 | 17.3 | -4.2 | 153 | 588 | - |
| 228B | Knyszyn | -23.87 | 2.39 | -21.48 | 5.90 | 3.2 | 46.8 | 17.2 | 2016 | 53.181 | 23.792 | 6.5 | 17.3 | -4.2 | 153 | 588 | - |
| 230B | Knyszyn | -24.65 | 2.39 | -22.26 | 5.27 | 3.2 | 46.6 | 17.1 | 2016 | 53.181 | 23.792 | 6.5 | 17.3 | -4.2 | 153 | 588 | - |
| 231B | Knyszyn | -25.45 | 2.39 | -23.05 | 3.63 | 3.5 | 46.2 | 15.6 | 2016 | 53.346 | 23.254 | 6.6 | 17.3 | -3.8 | 186 | 582 | - |
| 232B | Knyszyn | -25.50 | 2.39 | -23.11 | 2.58 | 3.2 | 46.3 | 16.8 | 2016 | 53.269 | 23.304 | 6.8 | 17.3 | -3.6 | 180 | 582 | - |
| 233B | Knyszyn | -24.63 | 2.39 | -22.24 | 5.17 | 3.2 | 46.7 | 16.9 | 2016 | 53.224 | 23.620 | 6.6 | 17.3 | -3.8 | 165 | 588 | - |
| 234B | Knyszyn | -23.53 | 2.39 | -21.13 | 4.21 | 3.2 | 43.2 | 15.7 | 2016 | 52.957 | 23.810 | 6.7 | 17.2 | -4.1 | 151 | 589 | - |
| 239B | Knyszyn | -24.63 | 2.39 | -22.23 | 4.27 | 3.2 | 46.4 | 16.8 | 2016 | 53.012 | 23.860 | 6.5 | 17.0 | -4.4 | 175 | 591 | - |
| 241B | Knyszyn | -24.49 | 2.39 | -22.09 | 6.50 | 3.4 | 47.8 | 16.5 | 2016 | 53.050 | 23.466 | 6.7 | 17.3 | -3.7 | 172 | 590 | - |
| 307B | Knyszyn | -25.90 | 2.01 | -23.89 | 2.12 | 3.2 | 47.1 | 17.0 | 2008 | 53.349 | 23.241 | 6.6 | 17.2 | -3.8 | 203 | 582 | - |
| 308B | Knyszyn | -24.28 | 2.10 | -22.18 | 5.83 | 3.3 | 47.5 | 17.0 | 2010 | 53.142 | 23.823 | 6.6 | 17.1 | -4.1 | 188 | 591 | - |
| 309B | Knyszyn | -25.46 | 2.01 | -23.45 | 0.65 | 3.3 | 46.9 | 16.6 | 2008 | 53.233 | 23.432 | 6.8 | 17.3 | -3.7 | 184 | 586 | - |
| 310B | Knyszyn | -24.39 | 2.06 | -22.33 | 3.56 | 3.3 | 47.1 | 16.7 | 2009 | 53.131 | 23.781 | 6.6 | 17.1 | -4.0 | 183 | 591 | - |
| 311B | Knyszyn | -24.92 | 2.10 | -22.82 | 3.48 | 3.4 | 47.4 | 16.1 | 2010 | 53.152 | 23.894 | 6.5 | 17.2 | -4.4 | 150 | 591 | - |
| 312B | Knyszyn | -24.15 | 2.10 | -22.04 | 3.02 | 3.4 | 46.2 | 15.8 | 2010 | 53.069 | 23.763 | 6.6 | 17.1 | -4.2 | 166 | 591 | - |
| 313B | Knyszyn | -24.98 | 2.10 | -22.88 | 0.61 | 3.4 | 45.5 | 15.5 | 2010 | 53.066 | 23.777 | 6.6 | 17.1 | -4.2 | 181 | 591 | - |
| 314B | Knyszyn | -22.86 | 2.13 | -20.74 | 5.82 | 3.4 | 46.7 | 15.8 | 2010/11 | 53.057 | 23.744 | 6.6 | 17.1 | -4.3 | 165 | 591 | - |
| 315B | Knyszyn | -25.25 | 2.10 | -23.14 | 1.16 | 3.6 | 46.0 | 14.7 | 2010 | 53.075 | 23.796 | 6.6 | 17.1 | -4.3 | 158 | 591 | - |
| 317B | Knyszyn | -24.72 | 2.06 | -22.67 | 0.96 | 3.6 | 46.5 | 14.9 | 2009 | 53.102 | 23.758 | 6.6 | 17.1 | -4.1 | 169 | 591 | - |
| 350B | Knyszyn | -24.63 | 2.10 | -22.52 | 7.61 | 3.2 | 46.6 | 16.9 | 2010 | 53.155 | 23.885 | 6.5 | 17.2 | -4.4 | 173 | 591 | - |
| 100P* | Piotrków | -23.99 | 2.34 | -21.64 | 4.04 | 3.5 | 45.6 | 15.1 | 2015 | 51.343 | 19.901 | 8.1 | 18.1 | -2.7 | 189 | 538 | - |
| 101P* | Piotrków | -25.01 | 2.34 | -22.66 | 3.74 | 3.4 | 45.1 | 15.7 | 2015 | 51.437 | 19.772 | 8.1 | 17.9 | -2.7 | 198 | 545 | - |
| 102P* | Piotrków | -24.69 | 2.34 | -22.35 | 3.44 | 3.4 | 45.3 | 15.4 | 2015 | 51.443 | 19.797 | 8.1 | 17.8 | -2.7 | 209 | 545 | - |
| 86P* | Piotrków | -24.71 | 2.39 | -22.31 | 4.51 | 3.3 | 41.9 | 14.8 | 2016 | 51.443 | 19.797 | 8.1 | 17.8 | -2.7 | 209 | 545 | - |
| 88P* | Piotrków | -25.00 | 2.34 | -22.66 | 1.56 | 3.3 | 44.7 | 15.9 | 2015 | 51.437 | 19.772 | 8.1 | 17.9 | -2.7 | 198 | 545 | - |
| 89PZ* | Piotrków | -24.96 | 2.39 | -22.57 | 4.77 | 3.4 | 45.2 | 15.6 | 2016 | 51.450 | 19.816 | 8.1 | 17.8 | -2.7 | 198 | 545 | - |
| 90P* | Piotrków | -24.94 | 2.34 | -22.59 | 3.13 | 3.3 | 44.0 | 15.5 | 2015 | 51.437 | 19.772 | 8.1 | 17.9 | -2.7 | 198 | 545 | - |
| 92P* | Piotrków | -24.56 | 2.34 | -22.21 | 4.58 | 3.6 | 43.1 | 14.2 | 2015 | 51.437 | 19.772 | 8.1 | 17.9 | -2.7 | 198 | 545 | - |
| 93P* | Piotrków | -25.35 | 2.34 | -23.01 | 3.38 | 3.6 | 43.8 | 14.2 | 2015 | 51.437 | 19.772 | 8.1 | 17.9 | -2.7 | 198 | 545 | - |
| 94P* | Piotrków | -25.37 | 2.39 | -22.98 | 2.17 | 3.3 | 44.9 | 15.7 | 2016 | 51.301 | 19.841 | 8.0 | 17.9 | -2.6 | 202 | 555 | - |
| 96P* | Piotrków | -24.81 | 2.34 | -22.47 | 2.87 | 3.4 | 43.4 | 14.7 | 2015 | 51.292 | 19.782 | 8.0 | 18.0 | -2.6 | 210 | 558 | - |
| 97P* | Piotrków | -25.32 | 2.34 | -22.98 | 2.59 | 3.4 | 43.8 | 15.1 | 2015 | 51.297 | 19.753 | 8.0 | 18.0 | -2.6 | 209 | 558 | - |
| 98PB* | Piotrków | -25.32 | 2.39 | -22.92 | 2.43 | 3.4 | 44.8 | 15.4 | 2016 | 51.300 | 19.932 | 8.0 | 17.9 | -2.8 | 186 | 555 | - |
| 99P* | Piotrków | -25.03 | 2.34 | -22.69 | 0.98 | 3.3 | 41.8 | 14.9 | 2015 | 51.333 | 19.809 | 8.0 | 17.8 | -2.6 | 187 | 558 | - |
| F1N* | Rum | -24.14 | 2.42 | -21.72 | 4.14 | 3.5 | 30.9 | 10.2 | 2016/17 | 57.005 | -6.336 | 8.7 | 13.6 | 4.5 | 223 | 1928 | 3 |
| F2B* | Rum | -24.14 | 2.42 | -21.71 | 4.06 | 3.9 | 32.7 | 9.9 | 2016/17 | 57.005 | -6.336 | 8.7 | 13.6 | 4.5 | 223 | 1928 | 3 |
| F3* | Rum | -23.85 | 2.42 | -21.43 | 4.18 | 3.5 | 32.9 | 10.9 | 2016/17 | 57.005 | -6.336 | 8.7 | 13.6 | 4.5 | 223 | 1928 | 3 |
| F4* | Rum | -24.14 | 2.42 | -21.72 | 3.65 | 3.8 | 28.8 | 8.8 | 2016/17 | 57.005 | -6.336 | 8.7 | 13.6 | 4.5 | 223 | 1928 | 3 |
| F5* | Rum | -23.97 | 2.42 | -21.55 | 4.05 | 3.5 | 34.1 | 11.4 | 2016/17 | 57.005 | -6.336 | 8.7 | 13.6 | 4.5 | 223 | 1928 | 3 |
| M10* | Rum | -23.70 | 2.42 | -21.28 | 4.47 | 3.7 | 34.7 | 11.0 | 2016/17 | 57.005 | -6.336 | 8.7 | 13.6 | 4.5 | 223 | 1928 | 3 |
| M6* | Rum | -23.40 | 2.42 | -20.98 | 3.77 | 3.8 | 24.7 | 7.5 | 2016/17 | 57.005 | -6.336 | 8.7 | 13.6 | 4.5 | 223 | 1928 | 3 |
| M7* | Rum | -24.28 | 2.42 | -21.86 | 3.79 | 3.7 | 26.4 | 8.3 | 2016/17 | 57.005 | -6.336 | 8.7 | 13.6 | 4.5 | 223 | 1928 | 3 |
| M8* | Rum | -23.75 | 2.42 | -21.33 | 3.68 | 3.5 | 30.3 | 10.2 | 2016/17 | 57.005 | -6.336 | 8.7 | 13.6 | 4.5 | 223 | 1928 | 3 |
| M9* | Rum | -24.38 | 2.42 | -21.96 | 2.62 | 3.5 | 31.4 | 10.3 | 2016/17 | 57.005 | -6.336 | 8.7 | 13.6 | 4.5 | 223 | 1928 | 3 |
| 115K* | Ustrzyki | -25.87 | 2.34 | -23.53 | 2.72 | 3.8 | 32.9 | 10.2 | 2015 | 49.485 | 22.457 | 7.2 | 16.7 | -3.0 | 460 | 802 | - |
| 119KP* | Ustrzyki | -24.87 | 2.34 | -22.52 | 2.94 | 3.5 | 48.2 | 16.0 | 2015 | 49.452 | 22.514 | 6.5 | 15.8 | -3.0 | 615 | 841 | - |
| 120K* | Ustrzyki | -25.85 | 2.34 | -23.50 | 3.23 | 3.7 | 30.3 | 9.6 | 2015 | 49.463 | 22.616 | 6.5 | 15.5 | -3.2 | 565 | 841 | - |
| 123K* | Ustrzyki | -25.23 | 2.34 | -22.89 | 3.18 | 3.6 | 31.5 | 10.1 | 2015 | 49.452 | 22.589 | 6.5 | 15.9 | -3.2 | 470 | 841 | - |
| 124KP* | Ustrzyki | -25.98 | 2.34 | -23.64 | 3.24 | 3.9 | 49.6 | 14.9 | 2015 | 49.293 | 22.383 | 6.0 | 15.9 | -2.9 | 624 | 933 | - |
| 126K* | Ustrzyki | -25.07 | 2.34 | -22.73 | 2.78 | 3.5 | 42.3 | 14.1 | 2015 | 49.343 | 22.583 | 6.5 | 15.6 | -3.0 | 537 | 841 | - |
| 128KP* | Ustrzyki | -25.25 | 2.34 | -22.91 | 2.98 | 4.0 | 47.1 | 13.6 | 2015 | 49.471 | 22.631 | 6.5 | 15.8 | -3.1 | 487 | 841 | - |
| 130KP* | Ustrzyki | -25.45 | 2.34 | -23.11 | 2.86 | 3.7 | 48.3 | 15.1 | 2015 | 49.549 | 22.594 | 6.8 | 15.8 | -3.0 | 554 | 778 | - |
| 167K* | Ustrzyki | -25.44 | 2.34 | -23.10 | 3.11 | 3.9 | 33.7 | 10.0 | 2015 | 49.670 | 22.469 | 7.7 | 17.2 | -2.6 | 394 | 691 | - |
| 168KP* | Ustrzyki | -26.33 | 2.34 | -23.99 | 2.43 | 3.8 | 43.5 | 13.5 | 2015 | 49.434 | 22.670 | 6.3 | 15.5 | -3.1 | 469 | 854 | - |
| 169KP* | Ustrzyki | -25.85 | 2.34 | -23.50 | 3.55 | 3.7 | 36.4 | 11.5 | 2015 | 49.463 | 22.629 | 6.5 | 15.8 | -3.1 | 495 | 841 | - |
| 171KP* | Ustrzyki | -26.00 | 2.34 | -23.66 | 2.50 | 3.9 | 42.3 | 12.5 | 2015 | 49.457 | 22.533 | 6.5 | 15.8 | -3.0 | 664 | 841 | - |
| 271K* | Ustrzyki | -24.59 | 2.45 | -22.15 | 3.51 | 3.5 | 32.9 | 11.0 | 2017 | 49.457 | 22.533 | 6.5 | 15.8 | -3.0 | 664 | 841 | - |
| 272K* | Ustrzyki | -24.92 | 2.45 | -22.47 | 3.24 | 3.5 | 32.4 | 10.7 | 2017 | 49.434 | 22.670 | 6.3 | 15.5 | -3.1 | 469 | 854 | - |
| 273K* | Ustrzyki | -25.68 | 2.45 | -23.23 | 2.40 | 3.6 | 33.3 | 10.9 | 2017 | 49.463 | 22.629 | 6.5 | 15.8 | -3.1 | 495 | 841 | - |
| 274K* | Ustrzyki | -25.46 | 2.45 | -23.01 | 3.23 | 3.7 | 32.5 | 10.2 | 2017 | 49.452 | 22.589 | 6.5 | 15.9 | -3.2 | 470 | 841 | - |
| 275K* | Ustrzyki | -25.40 | 2.45 | -22.95 | 3.11 | 3.9 | 32.7 | 9.8 | 2017 | 49.463 | 22.616 | 6.5 | 15.5 | -3.2 | 565 | 841 | - |
| 276K* | Ustrzyki | -25.98 | 2.45 | -23.53 | 2.13 | 3.7 | 32.5 | 10.2 | 2017 | 49.423 | 22.648 | 6.5 | 15.7 | -3.1 | 510 | 841 | - |
| 277K* | Ustrzyki | -25.43 | 2.45 | -22.99 | 2.90 | 3.6 | 33.3 | 10.8 | 2017 | 49.481 | 22.615 | 6.5 | 15.5 | -3.2 | 520 | 841 | - |
| 278K* | Ustrzyki | -25.57 | 2.45 | -23.12 | 2.61 | 3.8 | 33.2 | 10.2 | 2017 | 49.483 | 22.562 | 6.5 | 15.5 | -3.3 | 557 | 841 | - |
| 279K* | Ustrzyki | -25.33 | 2.45 | -22.88 | 2.66 | 3.7 | 30.5 | 9.8 | 2017 | 49.446 | 22.647 | 6.5 | 15.7 | -3.1 | 565 | 841 | - |
| 280K* | Ustrzyki | -25.81 | 2.45 | -23.37 | 2.52 | 3.9 | 33.2 | 9.9 | 2017 | 49.433 | 22.629 | 6.5 | 15.7 | -3.1 | 513 | 841 | - |
| 114SP* | Goleniów | -25.89 | 2.34 | -23.55 | 3.37 | 4.1 | 46.4 | 13.1 | 2015 | 53.449 | 14.846 | 8.6 | 18.2 | -0.9 | 20 | 564 | 26 |
| 131SP* | Goleniów | -25.79 | 2.34 | -23.44 | 6.55 | 3.9 | 44.5 | 13.2 | 2015 | 53.725 | 14.682 | 8.5 | 17.7 | -0.7 | 23 | 573 | 6 |
| 134SP* | Goleniów | -25.93 | 2.34 | -23.59 | 5.77 | 3.9 | 45.7 | 13.5 | 2015 | 53.449 | 14.846 | 8.6 | 18.2 | -0.9 | 20 | 564 | 25 |
| 135SP* | Goleniów | -25.44 | 2.34 | -23.10 | 3.94 | 4.0 | 46.9 | 13.6 | 2015 | 52.986 | 14.505 | 8.7 | 18.1 | -0.7 | 48 | 517 | 71 |
| 138SP | Goleniów | -24.48 | 2.34 | -22.14 | 4.52 | 3.4 | 47.6 | 16.5 | 2015 | 53.783 | 14.714 | 8.5 | 17.8 | -0.8 | 14 | 573 | 7 |
| 139SP* | Goleniów | -25.78 | 2.34 | -23.44 | 4.25 | 3.8 | 46.8 | 14.2 | 2015 | 53.815 | 14.805 | 8.5 | 17.7 | -0.8 | 15 | 573 | 13 |
| 141SP* | Goleniów | -25.63 | 2.34 | -23.29 | 4.13 | 4.2 | 48.0 | 13.3 | 2015 | 53.720 | 14.780 | 8.5 | 17.6 | -0.8 | 25 | 573 | 15 |
| 412SP | Goleniów | -25.34 | 2.50 | -22.84 | 5.58 | 3.5 | 45.9 | 15.5 | 2018 | 53.089 | 16.798 | 8.4 | 18.5 | -1.6 | 84 | 548 | 136 |
| 413SP | Goleniów | -24.76 | 2.50 | -22.26 | 4.05 | 3.3 | 46.7 | 16.6 | 2018 | 53.645 | 14.843 | 8.6 | 17.9 | -1.0 | 29 | 585 | 15 |
| 416SP | Goleniów | -24.32 | 2.50 | -21.81 | 1.10 | 3.4 | 48.8 | 17.0 | 2018 | 53.205 | 16.046 | 8.4 | 18.5 | -1.1 | 81 | 613 | 106 |
| 136SP* | Goleniów | -25.53 | 2.34 | -23.19 | 4.73 | 3.7 | 45.3 | 14.5 | 2015 | 53.804 | 14.899 | 8.5 | 17.8 | -0.8 | 19 | 597 | 16 |
| 137S* | Goleniów | -24.44 | 2.34 | -22.10 | 4.14 | 3.7 | 35.0 | 11.0 | 2015 | 53.454 | 14.998 | 8.6 | 18.3 | -1.1 | 48 | 564 | 39 |
| 143SP* | Goleniów | -24.49 | 2.34 | -22.15 | 3.07 | 4.3 | 43.4 | 11.8 | 2015 | 52.864 | 14.218 | 8.7 | 18.2 | -0.5 | 63 | 524 | 93 |
| 142SP | W. Pomerania | -25.92 | 2.34 | -23.58 | 5.17 | 3.5 | 47.2 | 15.9 | 2015 | 53.895 | 14.940 | 8.6 | 17.8 | -0.8 | 20 | 601 | 13 |
| 103S* | W. Pomerania | -25.81 | 2.34 | -23.46 | 4.40 | 4.1 | 41.9 | 11.9 | 2015 | 53.895 | 14.940 | 8.6 | 17.8 | -0.8 | 20 | 601 | 12 |
| 107S* | W. Pomerania | -23.71 | 2.34 | -21.37 | 6.31 | 3.9 | 36.8 | 10.9 | 2015 | 54.024 | 14.977 | 8.4 | 17.5 | -0.6 | 9 | 596 | 5 |
| 406SP | W. Pomerania | -25.21 | 2.50 | -22.71 | 5.34 | 3.4 | 47.0 | 16.1 | 2018 | 53.895 | 14.940 | 8.6 | 17.8 | -0.8 | 20 | 601 | 13 |
| 409SP | W. Pomerania | -25.12 | 2.50 | -22.62 | 4.44 | 3.4 | 46.7 | 15.9 | 2018 | 53.916 | 14.984 | 8.6 | 17.8 | -0.8 | 20 | 601 | 15 |
| 411SP | W. Pomerania | -24.80 | 2.50 | -22.30 | 4.44 | 3.5 | 46.6 | 15.8 | 2018 | 53.916 | 14.593 | 8.4 | 17.6 | -0.5 | 4 | 566 | 7 |
| 414SP | W. Pomerania | -24.95 | 2.50 | -22.45 | 5.35 | 3.4 | 45.0 | 15.5 | 2018 | 53.895 | 14.940 | 8.6 | 17.8 | -0.8 | 20 | 601 | 12 |
| 415SP | W. Pomerania | -25.30 | 2.50 | -22.80 | 5.00 | 3.4 | 44.9 | 15.3 | 2018 | 53.991 | 15.180 | 8.6 | 17.9 | -0.7 | 18 | 648 | 13 |
| 10CP* | Włodawa | -25.50 | 2.34 | -23.16 | 4.38 | 3.7 | 47.8 | 15.3 | 2015 | 51.444 | 23.128 | 7.6 | 18.0 | -3.4 | 186 | 522 | - |
| 15CP* | Włodawa | -25.09 | 2.34 | -22.74 | 3.01 | 3.7 | 45.2 | 14.2 | 2015 | 51.386 | 23.587 | 7.5 | 17.6 | -3.1 | 179 | 536 | - |
| 1CP* | Włodawa | -24.97 | 2.34 | -22.62 | 3.49 | 3.6 | 45.5 | 14.7 | 2015 | 51.504 | 23.484 | 7.5 | 17.7 | -3.2 | 183 | 527 | - |
| 20CP* | Włodawa | -25.25 | 2.34 | -22.91 | 3.36 | 3.7 | 46.6 | 14.7 | 2015 | 51.409 | 23.184 | 7.6 | 18.0 | -3.5 | 172 | 526 | - |
| 210CP* | Włodawa | -25.72 | 2.39 | -23.33 | 1.25 | 3.7 | 40.1 | 12.6 | 2016 | 51.502 | 23.404 | 7.5 | 17.7 | -3.1 | 181 | 527 | - |
| 211CP* | Włodawa | -24.99 | 2.39 | -22.60 | 3.36 | 3.5 | 46.6 | 15.6 | 2016 | 51.541 | 23.394 | 7.5 | 17.7 | -3.1 | 199 | 527 | - |
| 212CP* | Włodawa | -25.81 | 2.39 | -23.42 | 2.21 | 3.6 | 46.7 | 15.2 | 2016 | 51.500 | 23.505 | 7.5 | 17.8 | -3.4 | 176 | 528 | - |
| 214CP* | Włodawa | -25.20 | 2.39 | -22.80 | 3.90 | 4.0 | 42.1 | 12.3 | 2016 | 51.507 | 23.486 | 7.5 | 17.7 | -3.2 | 183 | 527 | - |
| 3CP* | Włodawa | -24.99 | 2.34 | -22.65 | 4.07 | 3.7 | 45.7 | 14.4 | 2015 | 51.502 | 23.404 | 7.5 | 17.7 | -3.1 | 181 | 527 | - |
| 4CP* | Włodawa | -25.17 | 2.34 | -22.82 | 4.01 | 3.8 | 45.7 | 13.9 | 2015 | 51.380 | 23.529 | 7.5 | 17.7 | -3.3 | 174 | 536 | - |
| 5CP* | Włodawa | -24.99 | 2.34 | -22.65 | 4.36 | 3.5 | 40.5 | 13.3 | 2015 | 51.386 | 23.587 | 7.5 | 17.6 | -3.1 | 179 | 536 | - |

**References**

1. Sykut M, Pawełczyk S, Borowik T, Pokorny B, Flajšman K, Niedziałkowska M. Intraindividual and interpopulation variability in carbon and nitrogen stable isotope ratios of bone collagen in the modern red deer (Cervus elaphus). Journal of Archaeological Science: Reports. 2020 2020/12/01/;34:102669.

2. Feng X. Long-term ci /ca response of trees in western North America to atmospheric CO2 concentration derived from carbon isotope chronologies. Oecologia. 1998 1998/11/01;117(1):19-25.
