# Supplementary material for: Environmental factors shaping stable isotope signatures of modern red deer (Cervus elaphus) inhabiting various habitats
Source: PLoS One. 2021 Aug 13;16(8):e0255398. doi: 10.1371/journal.pone.0255398 (PMC8362983; doi:10.1371/journal.pone.0255398)
Supplement: S4 Table — (DOCX) [file pone.0255398.s004.docx]

**Environmental factors shaping stable isotope signatures of modern red deer (*Cervus elaphus)* inhabiting various habitats**

Maciej Sykut*, Sławomira Pawełczyk, Tomasz Borowik, Boštjan Pokorny, Katarina Flajšman, Tjibbe Hunink, Magdalena Niedziałkowska

Corresponding author: Maciej Sykut mail: msykut@ibs.bialowieza.pl

S4 Table. The pairwise correlation matrix of following parameters: percentage share of forest cover, percentage share of open area, the mean annual temperature, the mean July temperature, the mean January temperature, the altitude, the annual precipitation and distance to seashore. Significant assays (*P* < 0.05) are given in bold.

| Parameter | Forest cover | Open area | Altitude  (m a.s.l.) | Annual precipitation (mm) | Annual mean temperature (°C) | January mean temperature (°C) | July mean temperature (°C) | Distance to seashore (km) |
| --- | --- | --- | --- | --- | --- | --- | --- | --- |
| Forest cover |  | **-1.00** | **0.26** | **-0.30** | **-0.60** | **-0.70** | **0.24** | **0.30** |
| Open area | **-1.00** |  | **-0.26** | **0.30** | **0.60** | **0.70** | **-0.24** | **-0.30** |
| Altitude  (m a.s.l.) | **0.26** | **-0.26** |  | **0.38** | **-0.33** | **-0.18** | **-0.40** | **0.60** |
| Annual precipitation (mm) | **-0.30** | **0.30** | **0.38** |  | **0.35** | **0.59** | **-0.51** | **-0.28** |
| Annual mean temperature (°C) | **-0.60** | **0.60** | **-0.33** | **0.35** |  | **0.80** | **0.15** | **-0.61** |
| January mean temperature (°C) | **-0.70** | **0.70** | **-0.18** | **0.59** | **0.80** |  | **-0.40** | **-0.59** |
| July mean temperature (°C) | **0.24** | **-0.24** | **-0.40** | **-0.51** | **0.15** | **-0.40** |  | -0.10 |
| Distance to seashore (km) | **0.30** | **-0.30** | **0.60** | **-0.28** | **-0.61** | **-0.59** | -0.10 |  |
