# Supplementary material for: Environmental factors shaping stable isotope signatures of modern red deer (Cervus elaphus) inhabiting various habitats
Source: PLoS One. 2021 Aug 13;16(8):e0255398. doi: 10.1371/journal.pone.0255398 (PMC8362983; doi:10.1371/journal.pone.0255398)
Supplement: S5 Table — (DOCX) [file pone.0255398.s005.docx]

**Environmental factors shaping stable isotope signatures of modern red deer (*Cervus elaphus)* inhabiting various habitats**

Maciej Sykut*, Sławomira Pawełczyk, Tomasz Borowik, Boštjan Pokorny, Katarina Flajšman, Tjibbe Hunink, Magdalena Niedziałkowska

Corresponding author: Maciej Sykut mail: msykut@ibs.bialowieza.pl

S5 Table. Statistical differences (Kruskal-Wallis test) between *δ*^13^C (above the diagonal) and *δ*^15^N (below the diagonal) values in bone collagen of red deer inhabiting different study sites. Significant assays (*P* < 0.05) are given in bold.

| Study site | Augustów | Bardo | Białowieża | Chełm | Dukla | Flevoland | G. Pomerania | Hru & Jav | Knyszyn | Piotrków | Rum | Ustrzyki | Goleniów | W.Pomerania | Włodawa |
| --- | --- | --- | --- | --- | --- | --- | --- | --- | --- | --- | --- | --- | --- | --- | --- |
| Augustów |  | 1.000 | 1.000 | 0.184 | 1.000 | 0.341 | 1.000 | 1.000 | 1.000 | 1.000 | **0.015** | 1.000 | 1.000 | 1.000 | 1.000 |
| Bardo | 1.000 |  | 1.000 | 1.000 | 1.000 | 1.000 | 1.000 | 1.000 | 1.000 | 1.000 | 0.119 | 0.585 | 1.000 | 1.000 | 1.000 |
| Białowieża | 1.000 | 1.000 |  | **<0.001** | 1.000 | **0.002** | 0.261 | 1.000 | 1.000 | 1.000 | **<0.001** | 1.000 | 1.000 | 1.000 | 1.000 |
| Chełm | 0.054 | 1.000 | 0.186 |  | **<0.001** | 1.000 | 1.000 | 1.000 | 1.000 | 1.000 | 1.000 | **<0.001** | 0.058 | 1.000 | **0.028** |
| Dukla | 1.000 | 1.000 | 1.000 | **<0.001** |  | **0.003** | 0.286 | 1.000 | 1.000 | 1.000 | **<0.001** | 1.000 | 1.000 | 1.000 | 1.000 |
| Flevoland | **<0.001** | **0.004** | **<0.001** | 1.000 | **<0.001** |  | 1.000 | 1.000 | 1.000 | 1.000 | 1.000 | **<0.001** | 0.115 | 1.000 | 0.057 |
| G. Pomerania | 1.000 | 1.000 | 1.000 | **0.002** | 1.000 | **<0.001** |  | 1.000 | 1.000 | 1.000 | 0.620 | **0.008** | 1.000 | 1.000 | 1.000 |
| Hru & Jav | 1.000 | 1.000 | 1.000 | **0.045** | 1.000 | **<0.001** | 1.000 |  | 1.000 | 1.000 | 0.248 | 1.000 | 1.000 | 1.000 | 1.000 |
| Knyszyn | 1.000 | 1.000 | 1.000 | 1.000 | 1.000 | **0.003** | 1.000 | 1.000 |  | 1.000 | 0.124 | 0.112 | 1.000 | 1.000 | 1.000 |
| Piotrków | 1.000 | 1.000 | 1.000 | 0.337 | 1.000 | **<0.001** | 1.000 | 1.000 | 1.000 |  | 0.134 | 0.976 | 1.000 | 1.000 | 1.000 |
| Rum | 1.000 | 1.000 | 1.000 | 1.000 | 0.904 | 0.377 | 1.000 | 1.000 | 1.000 | 1.000 |  | **<0.001** | **0.005** | 0.168 | **0.002** |
| Ustrzyki | 1.000 | 1.000 | 1.000 | **0.003** | 1.000 | **<0.001** | 1.000 | 1.000 | 1.000 | 1.000 | 1.000 |  | 1.000 | 1.000 | 1.000 |
| Goleniów | 1.000 | 1.000 | 1.000 | 1.000 | 0.059 | 0.963 | 0.165 | 0.952 | 1.000 | 1.000 | 1.000 | 0.237 |  | 1.000 | 1.000 |
| W. Pomerania | 0.068 | 1.000 | 0.221 | 1.000 | **0.002** | 1.000 | **0.006** | **0.050** | 1.000 | 0.286 | 1.000 | **0.009** | 1.000 |  | 1.000 |
| Włodawa | 1.000 | 1.000 | 1.000 | 1.000 | 1.000 | **0.014** | 1.000 | 1.000 | 1.000 | 1.000 | 1.000 | 1.000 | 1.000 | 1.000 |  |
