# Supplementary figures and images for: Environmental factors shaping stable isotope signatures of modern red deer (Cervus elaphus) inhabiting various habitats
Source: PLoS One. 2021 Aug 13;16(8):e0255398. doi: 10.1371/journal.pone.0255398 (PMC8362983; doi:10.1371/journal.pone.0255398)

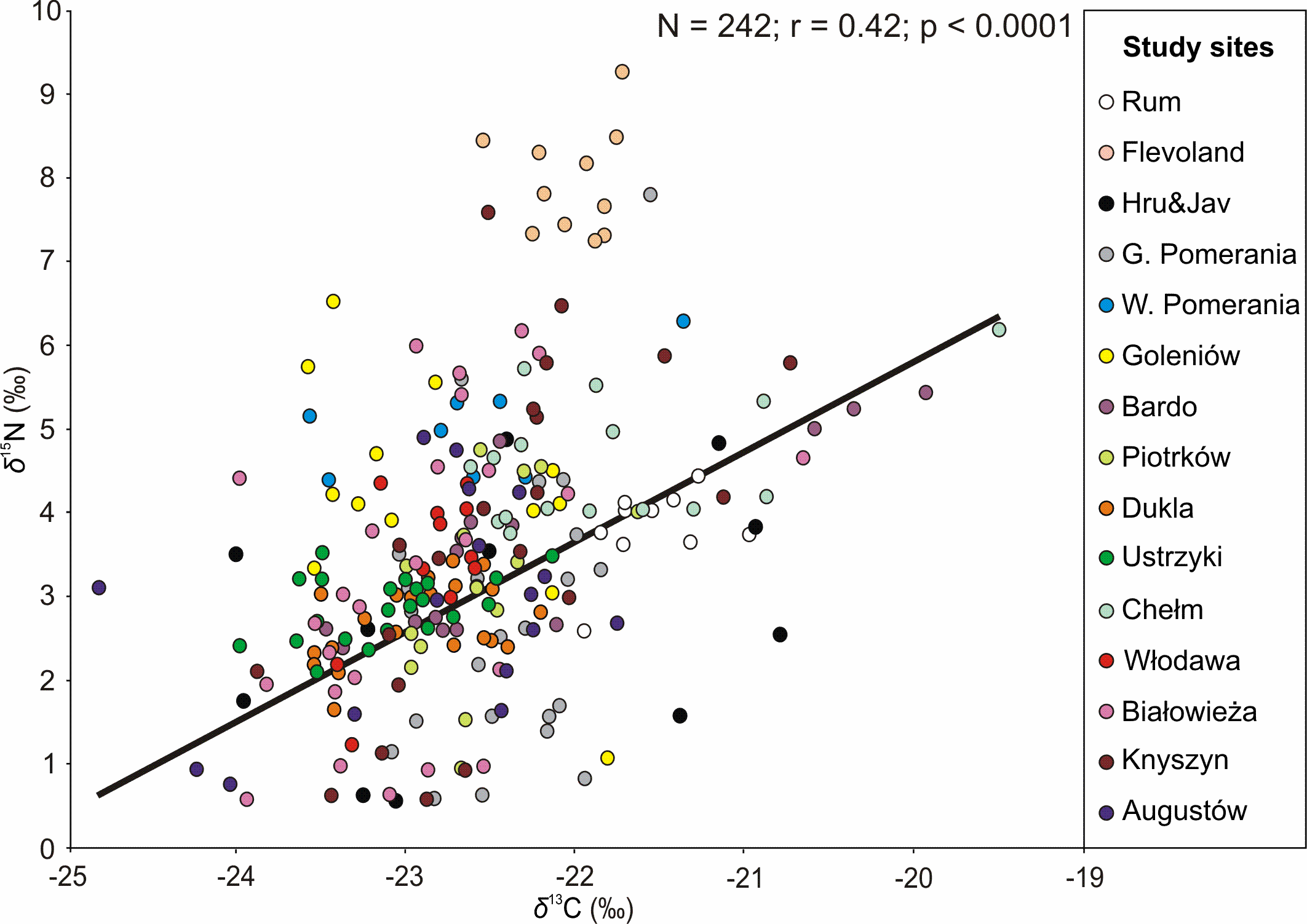

Supplement: S1 Fig — (TIF) [file pone.0255398.s006.tif]
